# Differential tetraspanin genes expression and subcellular localization during mutualistic interactions in *Phaseolus vulgaris*

**DOI:** 10.1371/journal.pone.0219765

**Published:** 2019-08-22

**Authors:** Saul Jimenez-Jimenez, Olivia Santana, Fernando Lara-Rojas, Manoj-Kumar Arthikala, Elisabeth Armada, Kenji Hashimoto, Kazuyuki Kuchitsu, Sandra Salgado, Jesús Aguirre, Carmen Quinto, Luis Cárdenas

**Affiliations:** 1 Departamento de Biología Molecular de Plantas, Instituto de Biotecnología, Universidad Nacional Autónoma de México, Cuernavaca, Morelos, México; 2 Ciencias Agrogenómicas, Escuela Nacional de Estudios Superiores Unidad León-Universidad Nacional Autónoma de México, León, Guanajuato, México; 3 Department of Applied Biological Science, Tokyo University of Science, Yamazaki, Noda, Chiba, Japan; 4 Instituto de Fisiología Celular, Universidad Nacional Autónoma de México, Ciudad de México, México; Estacion Experimental del Zaidin, SPAIN

## Abstract

Arbuscular mycorrhizal fungi and rhizobia association with plants are two of the most successful plant-microbe associations that allow the assimilation of P and N by plants, respectively. These mutualistic interactions require a molecular dialogue, i.e., legume roots exude flavonoids or strigolactones which induce the Nod factors or Myc factors synthesis and secretion from the rhizobia or fungi, respectively. These Nod or Myc factors trigger several responses in the plant root, including calcium oscillations, and reactive oxygen species (ROS). Furthermore, superoxide and H_2_O_2_ have emerged as key components that regulate the transitions from proliferation to differentiation in the plant meristems. Similar to the root meristem, the nodule meristem accumulates superoxide and H_2_O_2_. Tetraspanins are transmembrane proteins that organize into tetraspanin web regions, where they recruit specific proteins into platforms required for signal transduction, membrane fusion, cell trafficking and ROS generation. Plant tetraspanins are scaffolding proteins associated with root radial patterning, biotic and abiotic stress responses, cell fate determination, and hormonal regulation and recently have been reported as a specific marker of exosomes in animal and plant cells and key players at the site of plant fungal infection. In this study, we conducted transcriptional profiling of the tetraspanin family in common bean (*Phaseolus vulgaris L*. var. Negro Jamapa) to determine the specific expression patterns and subcellular localization of tetraspanins during nodulation or under mycorrhizal association. Our results demonstrate that the tetraspanins are transcriptionally modulated during the mycorrhizal association, but are also expressed in the infection thread and nodule meristem development. Subcellular localization indicates that tetraspanins have a key role in vesicular trafficking, cell division, and root hair polar growth.

## Introduction

The symbiotic interaction between rhizobia and legumes requires a molecular dialogue that involves the exchange of specific signaling molecules. Legumes secrete particular flavonoids or strigolactones that are specifically recognized by rhizobia or mycorrhizal fungi [1]. These compounds induce the expression of specific genes, which encode proteins involved in the synthesis and secretion of Nod factors (NFs) from rhizobia, which are lipochitin-oligosaccharides or the Myc factor from the mycorrhizal fungi [2, 3]. Thereafter, NFs or Myc factors are specifically recognized by the plant root and induce several responses, including ionic changes, membrane depolarization, cytoskeleton rearrangements, reactive oxygen species (ROS) generation, and gene expression [4–7]. Soon after the rhizobia reach the root hair, microfilament structures and Ca^2+^ gradients undergo dramatic changes in the tip region of root hair cells in response to NFs [8–11]. In *Arabidopsis thaliana* root hairs, ROS-mediated Ca^2+^ channel activity supports polar growth [12–14]. Therefore, ionic responses, ROS production, and cytoskeletal rearrangements have been suggested as key players in reprogramming root hair growth, which allows the root hair tip curling response during the establishment of symbiotic interactions in legume plants. Then, a tunnel-like infection thread (IT) forms through invagination of the plasma membrane and the cell wall [2]. While the bacteria travel inside the IT through the root hair, the cortical cells divide in a NF-dependent manner to form the nodule primordia that the rhizobia colonize in structures named symbiosome. Once mature, the nodule is able to fix atmospheric nitrogen [15]. On the other hand, the arbuscular mycorrhizal association induces the hypopodium and the further invasion of the cortical cells, which end up with the arbuscule formation. Both processes, the bacterial colonization trough the infection thread and arbuscule formation, require an active vesicular trafficking, endocytosis and exocytosis in order to increase the membranal surface required for symbiosome and arbuscule formation [16, 17].

Given their wide distribution in mammals, insects, fungi, mosses, and plants, tetraspanins likely coemerged in multicellular organisms during evolution [18]. In animal cells, tetraspanins are typically localized at the cell–cell interface in tetraspanin-enriched microdomains (TEMs), where they associate with each other and with other membrane-bound molecules and build important molecular platforms or cell–cell interactions [19, 20]. Tetraspanins get their name from their four transmembrane domains. The N and C tails of tetraspanins are localized on the cytoplasmic side of the membrane, and four transmembrane domains allow for the formation of two extracellular loops, one small and one large. The large loop has highly conserved cysteine residues, which could act as redox and pH sensors or promote protein–protein interactions. The small loop in plant tetraspanins contains a cysteine residue that is absent in animal tetraspanins [21]. Although tetraspanins in animal cells have been involved in various biological functions, such as cell motility, morphology, signaling, plasma membrane dynamics, and protein trafficking, how tetraspanins engage in plant cells functions at the cellular level is largely unknown [18]. Furthermore, tetraspanin have been described in animal and recently in plant cells as key components and specific markers of the exosomes, which are vesicles derived from the exocytic multivesicular bodies (MVB) that carry important molecules such as lipids, proteins, messenger RNA, and microRNAs, that play important roles in cell-to-cell communication in animal [22]. In plant cells it has been recently reported that exosomes go beyond organism boundaries and inhibit a pathogenic interaction in plants [23]. There is also an emerging idea that tetraspanins are part of a mechanism that generates ROS [24, 25]. In animal cells, H_2_O_2_ has been described as an important component of axonal regeneration after acute injury [26]. However, the injured neurons do not express the NADPH oxidase required for ROS generation. Instead, macrophages recruited to the vicinity secrete exosomes carrying NOX enzymes, which are taken up by the injured neuron via endocytosis and promote axonal growth in a ROS-dependent manner with a key participation of exosomes [26–28]. On the other hand, the cuticle exoskeleton of the nematode *Caenorhabditis elegans*, in a similar way to the cell wall in plant cells, requires the ROS-dependent cross-linking of tyrosine residues, in a process assisted by BLI-3, which is a DUOX NADPH oxidase, and tetraspanin TSP-15 [24, 25, 29, 30]. When plants are infected with the pathogenic fungus *Magnaporthe grisea*, the fungal tetraspanin Pls1 and an NADPH oxidase are localized at the infection sites or appressorium structure to generate a ROS accumulation response that is required to reestablish the appressorium polarity [24, 25, 31–33]. Therefore, accumulating evidence links tetraspanin with ROS and polarity. Furthermore, tetraspanins have been identified at the tip of growing pollen tubes, a region that requires NADPH oxidase activity for the generation of ROS, a key player regulating polar growth [34]. However, we do not know if tetraspanins also function in pollen tube or even root hair polar growth [35].

Tetraspanins also accumulate in the female gametophyte, suggesting that they have an active role during gametophyte development or fertilization, a well-described ROS-dependent process. Furthermore, tetraspanins function during the transition from the floral meristem to the gynoecium as well as during the somatic-to-reproductive cell fate transition during megasporogenesis, suggesting that these proteins are regulators of cell fate determination [35–37]. As tetraspanins are also expressed in specialized tissues, such as the the quiescent center or the early initial cells that give rise to lateral roots meristems, these proteins may function in specific tissues or contribute to cell fate determination [18, 35, 38]. The meristematic distribution of some tetraspanins suggests that these proteins might be involved in regulating meristematic activity, which is highly dependent on ROS accumulation generated by NADPH oxidase activity, with superoxide-promoting meristematic activity and H_2_O_2_-promoting cell differentiation [39, 40].

On the other hand, NADPH-oxidase-derived ROS is a key component during nodule development and mycorhizal formation [41–43]. Since superoxide and H_2_O_2_ are produced during nodulation [43], it is possible that the molecular mechanism that maintains meristematic activity in the root is conserved during nodule meristem development [38]. Therefore, it is plausible that the ROS requirement for meristematic activity in the plant root is conserved during primordium development during nodule organogenesis and that tetraspanins have similar functions during the early stages of nodule development, as described in lateral root formation in *Arabidopsis* [38].

Here, we report the differential expression profile of the tetraspanin family in *P*. *vulgaris* in response to rhizobia or NF inoculation in several specific plant cells, such as root primordia, root hairs, and nodules at several developmental stages. *PvTET8*, *PvTET4* and *PvTET3* were highly expressed in the root meristematic region and during the early stages of primordium nodule development. Since PvTET8 was highly induced during nodulation, but not during mycorrhizal association, we suggest that this tetraspanin plays a particular role during nodulation, including the infection thread formation. Furthermore, the subcellular localization of some tetraspanins at the apical plasma membrane of *P*. *vulgaris* root hairs and in cytoplasmic vesicles suggests that these proteins function in polar growth, a central process during infection thread formation and migration. Our findings suggest that tetraspanins may also contribute to the vesicular trafficking required for localized exocytosis during the infection process or contribute to the exosome biogenesis as described during the pathogenic responses.

## Materials and methods

### Phylogenetic analysis

Using the methodology described by Huang et al. (2010), tetraspanin sequences containing four transmembrane domains were database screened, including a small and large extracellular loop, using bioinformatics tools. The sequences were aligned in ClustalW. To avoid subjective bias, manual alignment editing was minimized. A phylogenetic tree was built using MEGA version 6.0.6 with 1000 bootstrap tests and pairwise deletion.

### Vector construction for analyzing the activity promoter and subcellular localization

To evaluate promoter activity, the *pPvTET1A*::*GFP-GUS*, *pPvTET8*::*GFP-GUS and pPvTET3*::*GFP-GUS* construct, which includes the 1000-bp fragment upstream of the initiation codon of TET1A, TET8 and TET3 respectively, was created by PCR using *P*. *vulgaris* cv *Negro Jamapa* genomic DNA as template and gene-specific primers (S1 Fig). Each product of PCR was cloned into the pENTR/D-TOPO vector (Invitrogen) and recombined into the destination binary vector pBGWFS7.0 using Gateway LR Clonase II Enzyme Mix (Invitrogen) [44]. In each step, the presence of the insert was confirmed by Sanger sequencing and PCR. All constructed plasmids were introduced by electroporation into *Agrobacterium rhizogenes* strain K599. To design constructs for overexpressing *PvTET10*, *PvTET6* and *PvTET3*, the open reading frame of each gene was isolated from *P*. *vulgaris* cDNA and inserted into the pH7WG2D.1 binary vector under the control of the constitutive 35S promoter [45]. Empty pH7WG2D.1 vector, which constitutively expresses GFP, was used as the control in the overexpression system. All constructed plasmids were introduced by electroporation into *A*. *rhizogenes* strain K599 and used for further generation of transgenic roots or *Agrobacterium tumefaciens* strain AGL1 for transient expression in *N*. *benthamiana* leaves.

### Bean hairy root transformation

Common bean seeds (*Phaseolus vulgaris cv* Negro Jamapa) were used for *A*. *rhizogenes* K599-mediated transformation to generate hairy roots harboring a construct of interest in composite plants using a previously described method [46].

### Treatment with Nod factors by nebulization

Common bean seedlings were incubated with Nod factors at 48 h post germination (hpg) by nebulization with the Omron ComAir Nebulizer System Model NE-C801 according to the manufacturer’s instructions. The Nod factors were purified according to our reported method [47]. A kinetic of transcript accumulation was conducted in roots incubated with Nod factors at 12, 24, and 36 h post inoculation for each tetraspanin. Nebulization with 1% CHAPS (w/v) was used as a control.

### Nodulation assays

*Rhizobium tropici* CIAT899 bacteria were grown in 250-mL flasks containing 100 mL of PY broth supplemented with 7 mM CaCl_2_, 50 μg/mL rifampicin, and 20 μg/mL nalidixic acid, in a shaking incubator (250 rpm) at 30°C until the suspension reached an OD_600_ of 0.8. For nodulation assays, transgenic composite plants were transplanted under hydroponic conditions in glass tubes containing Fahreus medium and inoculated with 1 mL of a *R*. *tropici* CIAT899 suspension diluted to an OD_600_ of 0.05 in 10 mM MgSO_4_ and grown in a controlled environment chamber (16 h light/8 h darkness, at 26°C). At the indicated time points after inoculation, inoculated roots were frozen in liquid nitrogen and stored at −80°C. At 5 and 7 days post infection (dpi), the root region close to the tail that is most susceptible to nodule formation was selected, and at 10, 14, and 18 dpi, only nodules were selected. In all cases, the equivalent root region of uninoculated roots was collected as a control.

### Mycorrhizal spore inoculation and mycorrhization

Common bean seedlings at 48 hpg were transferred into pots (20-cm diameter) with vermiculite that was previously well inoculated in the pot with 1 gr of *Rhizophagus irregularis* substrate containing on average 800 spores homogeneously distributed. Inoculated plants were irrigated twice weekly with half-strength B&D solution containing a low concentration of potassium phosphate (50 μM; a 95% reduction compared with the control (500μM) to potentiate AM colonization [48]). As controls, two conditions without spores of *R*. *irregularis* were used: one set of plants were irrigated with 50 μM potassium phosphate (scarcity phosphate) and the other with 500 μM potassium phosphate (standard condition). AM fungal colonization status was determined by light and confocal microscopy, as indicated. Root samples were collected at 1, 2, 3, and 6 wpi and frozen in liquid nitrogen. All samples were stored at −80°C.

### Quantification of transcript levels by RT-qPCR analysis

Total RNA was extracted from frozen tissues using TRIzol reagent (Life Technologies) according to the manufacturer’s instructions. To eliminate contaminating genomic DNA, total RNA samples (1 μg in 20 μL) were treated with 1 unit of DNaseI (RNase-free; Invitrogen) at 37°C for 30 min and then at 65°C for 10 min. Two-step RT-qPCR was performed using Maxima SYBR Green qPCR Master Mix (2X; Thermo Fisher Scientific), following the manufacturer’s instructions. Each reaction was set up using 100 ng of cDNA as template in a 20-μL final volume. Gene-specific primers used in RT-qPCR reactions are listed in S1 Fig. qPCRs were performed in a LightCycler 480 real-time PCR system (Roche). Relative transcript abundance was calculated using the formulae reported by Schmitteng et al. [49]. *P*. *vulgaris* Elongation Factor 1α (Pv-EF1α) was used as a reference gene, as previously described. RT-qPCR data are averages of three independent experiments or biological replicates with two technical replicates.

### Root hair and root isolation

The roots of *P*. *vulgaris* seedlings were divided and separated at 48 hpg. First, the region of the primary root that contained root hairs was cut into three equal segments named zones I, II, and III. Zone I contained the tip of the root that contained the initial or bulging out root hairs, zone II contained the rapidly growing root hairs, and zone III contained the mature or full-grown root hairs. Each fraction was collected in different containers in liquid nitrogen. Samples were stirred vigorously to separate the root hairs from the roots. Root hairs were isolated by pouring the liquid nitrogen mixture through a metal strainer. At the end, the root hairs were separated from the root (now shaved root) using a strainer. The shaved roots corresponding to regions I, II, and III were collected for tetraspanin transcript analysis. Fractions were stored at −80°C until use. In each biological replicate, in order to confirm the different developmental stage of root hairs, the level of *RabA2* transcript was measured to assess the differential expression of this gene in each enriched tissue fraction.

### Subcellular localization of common bean tetraspanins

Transient expression assays were conducted in *Nicotiana benthamiana* leaves to determine the subcellular localization of PvTET3 and PvTET6 proteins. The molecular construction carrying 35S:PvTET3-GFP and 35S:PvTET6-GFP was transferred to *A*. *tumefaciens* AGL1. For transient assays, leaves from 4- to 6-week-old wild-type *N*. *benthamiana* plants were coinfiltrated with the agrobacterium suspension harboring 35S:PvTETx-GFP. The infiltrated plants were marked and kept in a growth room at 16 h light/8 h darkness at 25 ± 2°C. Plasmolysis was induced using a 1M NaCl hypertonic solution. Fluorescence was visualized 48–72 h after infiltration using a spinning disk confocal microscope (Yokogawa, Japan) as described below.

### Microscopy imaging and analysis

Transgenic roots were mounted in chambers adapted from large Petri dishes with a hole in the center. The hole was covered with a large glass coverslip. The chamber contained a layer of solid Fahreus medium (with Phytagel at 0.8%) and cellophane paper to prevent root movement. Roots were visualized under the inverted microscope (Nikon Eclipse Ti-E, Japan) with a 40x/1.25 NA water immersion lens (Nikon). For confocal images, we used a spinning disk confocal system (Intelligent Imaging Innovations /3i, USA) consisting of a CSU-W1 confocal head (Yokogawa, Japan) and a modular solid-state laser stack; Slidebook software was used to control the system and capture images (Intelligent Imaging Innovations /3i, USA). Images were recorded with a digital camera (Andor-IXON 3; Andor^TM^ Technology) for 1–2 min at 512-nm resolution and with frame rates of 100–300 ms. GFP fluorescence was obtained by exciting with an argon/2 ion laser (488 nm), and emitted fluorescence was collected using an emission filter (500 to 530 nm).

### Statistical analysis

Data processing and statistical analysis were performed using GraphPad Prism version 6.00 for Windows (GraphPad Software). An unpaired two-tailed Student’s *t*-test was used to determine whether data from two different groups were significantly different, Double, or triple asterisks above the columns indicate differences that are statistically significant (p-value < 0.05).

## Results

### Plant tetraspanins have a unique cysteine residue in the small extracellular loop

**Fig 1:**
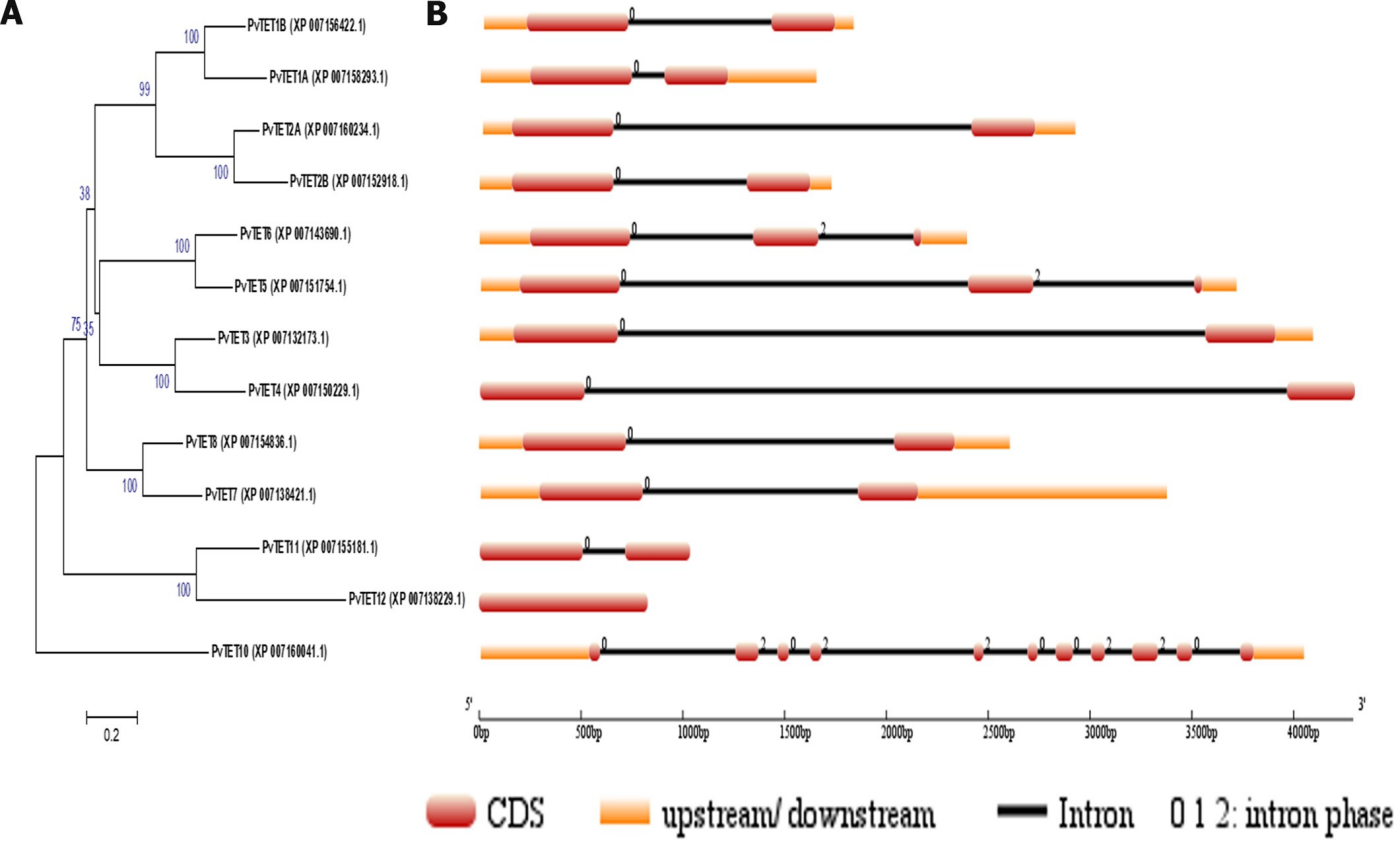
A rooted neighbor-joining phylogenetic tree of common bean tetraspanin. (A) On the left shown is the phylogenetic tree constructed using MEGA6.06, with amino acidic sequences from phytozome.org. Numbers above branches indicate bootstrap percentage values. PvTET proteins were clustered based on a significant bootstrap value of ≥50%. (B) On the right using an online tool, Gene Structure Display Server (GSDS; http://gsds.cbi.pku.edu.cn/), was used to draw the tetraspanin gene structure. Red boxes indicate exons, black lines depict introns, upstream/downstream sequences are shown as oranges boxes. Intron phases are indicated at exon–intron junctions.

To determine the number of tetraspanin members in common bean (*Phaseolus vulgaris L*. var. Negro Jamapa), we searched the database of nonredundant protein sequences in NCBI using BLASTP (Protein-protein BLAST) using AtTET10 as query. AtTET10 and OsTET14 from *O*. *sativa* can be considered the founders of the tetraspanin family in their respective species [35, 50]. We identified 13 putative tetraspanin sequences in *P*. *vulgaris*, 25 in *Glycine max*, 9 in *Medicago truncatula*, and 5 in *Lotus japonicus* (Fig 1 and S2 Fig). Seventeen tetraspanins have been described in the model plant *Arabidopsis* and 15 in *Oryza sativa* [35, 50]. PvTET10, and other orthologs of AtTET10 and OsTET14, have between 10 and 12 introns in all reported genomes, including *Marchantia polymorpha* and *Physcomitrella patens*.

The amino acid sequences and functional motifs previously described in *Arabidopsis* allowed us to select tetraspanin members in *P*. *vulgaris* and compare them to other tetraspanins in the legume genome database reported in Phytozome (https://phytozome.jgi.doe.gov/pz/portal.html). Next, we conducted a bioinformatic screen based on several sequences, including the transmembrane domains, the cysteine pattern in the large extracellular loop (LEL), the single cysteine residue located exclusively in plant tetraspanins in the small extracellular loop (SEL), and the GCC(K/R)P signature in the large extracellular loop. Multiple sequence alignment and motif analyses revealed that common bean tetraspanins maintain the general features described for plant tetraspanins, with high conservation in specific motifs. For instance, in *P*. *vulgaris*, these proteins share an average of 38% identity and 42% similarity, which is comparable to that observed for *O*. *sativa* and *Arabidopsis*.

Based on these data, we generated an unrooted phylogenetic three using PvTET10 as a query from *Arabidopsis* (S2 Fig and S3 Fig). In our analysis, PvTET10 clustered with AtTET10 and OsTET14, whereas PvTET1A and PvTET1B clustered with AtTET1 (TORNADO) and AtTET2, and PvTET8 clustered with OsTET7-9 and AtTET8-9. We did not find a homolog of AtTET13 in the *P*. *vulgaris* genome or in any other legume genome. This could suggest the presence of a particular clade in *Arabidopsis* or in the Brassicaceae that does not exist in other plants, at least not in *P*. *vulgaris* or in legumes. In our molecular tree, we identified 7 groups, each of which contained at least two members, except the group that contained PvTET10 alone. Sequence identity and similarity analysis coupled with the number of introns supported the idea that PvTET10 is a common ancestor of tetraspanin in common bean, as described for its homologs in *Arabidopsis* (Fig 1). By contrast, PvTET12, which has no introns, is the most recent tetraspanin member and arose by functional divergence and loss of introns [51, 52].

GmTET12A (accession number XP_003551251.1) and GmTET12B (accession number XP_003547633.1) are homologs of PvTET12 (accession number XM_007138229.1). In *M*. *truncatula*, we identified one homologue of TET12 (accession number XP_013463988.1); however, we did not find a corresponding homolog in *L*. *japonicus*. The cytoplasmic N and C termini of plant tetraspanins each have 6–10 aa, making them shorter than animal tetraspanins, which contain between 9 and 40 aa, and even shorter than fungal ones, which contain between 4 and 100 aa.

In addition, we conducted an exhaustive *in silico* characterization of tetraspanins in common bean to predict disulfide bond formation. We used a bioinformatic tool for disulfide connectivity prediction named DiANNA (clavius.bc.edu/~clotelab/DiANNA/) and found that disulfide bonds form between the single cysteine located in the SEL and the cysteine residues found in the LEL of all common bean tetraspanins, as previously suggested for tetraspanins in *Arabidopsis* [35].

### Tetraspanins PvTET3 and PvTET1A are highly expressed in root and root hair cells

**Fig 2:**
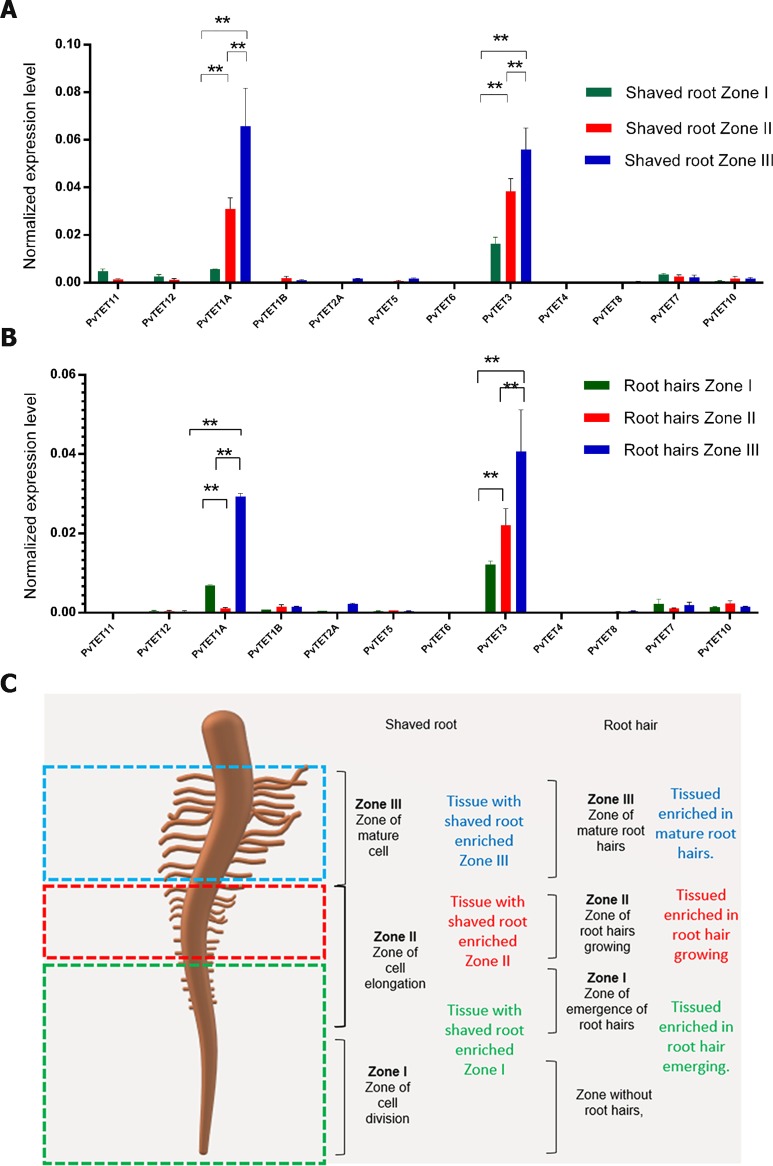
Tetraspanin gene expression profile in *P*. *vulgaris* root or root hairs from different developmental stages. The relative expression of each *PvTET* gene was evaluated by qRT–PCR in three different sections of root at 48 h post germination (hpg). Mature zone or Zone III (blue), elongation zone or Zone II (red), and meristematic, elongating and differentiating region or Zone I (green). Transcript accumulation was normalized to the expression of *EF1a*, which was used as a reference gene. Bars represent means ± SEM from at least three independent biological replicates with three technical repeats. (A) Tetraspanin (TET) gene expression in shaved root and (B) root hairs at different development stages at 48 hpg. Tissues enriched with emerging or bulging root hairs from Zone I (green bar), tissues enriched with growing root hairs (red bar) and tissues enriched with mature root hairs (blue bar). Transcript accumulation was normalized to the expression of *EF1a*, which was used as a reference gene. Bars represent means ± SEM from at least three independent biological replicates with three technical repeats. P-values <0.05 are marked with two asterisks (Student’s t-test). (C) Cartoon depicting the different root and root hairs zones analyzed.

To establish the transcript accumulation for the different tetraspanin genes in *P*. *vulgaris* roots, we selected root tissues and sectioned different regions with different developmental stages according to the experimental requirements (described in Materials and methods). In our experiments with *P*. *vulgaris*, we considered the three developmental root hair zones, which were evident by 48 h post germination (hpg). As depicted in Fig 2C we sectioned the three different root zones and separated the root hair cells from the root tissue, generating the enriched root hair fraction from the three developmental stages (zones I, II, and III) and the corresponding shaved root (Fig 2C). Transcript accumulation for all tetraspanin genes, except *PvTET2B* due to the lack of specific regions for primers design for amplification (accession number XP 007152918.1), was determined in each one of the generated samples (root hairs and shaved root); this analysis was conducted by selecting each tetraspanin family member and determining its accumulation in root hairs at different developmental stages (Fig 2B).

The monomeric GTPase PvRabA2 is a good marker of the different developmental stages of root hairs due to its key role in root hair tip growth (S4 Fig), although it is also expressed in root tissue [53, 54]. We observed that RabA2 transcript accumulated in root hairs; as expected, the transcript accumulation was higher in zones II and III, where the cells are rapidly expanding or reaching full length, compared with zone I, where root hairs are just bulging out (S4 Fig). As expected, the shaved root section that lacked root hairs also had increased *PvRabA2* transcript accumulation in zones I, II, and III, again with higher accumulation in zones II and III (S4 Fig). Therefore, these root sections were used to determine the transcript accumulation for selected tetraspanin members in *P*. *vulgaris*. We found that *PvTET3* and *PvTET1A* are more abundant in shaved roots or root hairs from zones II and III than from zone I (Fig 2A and 2B). However, *PvTET1A* present a very low expression in root hairs from zone II, but it is expressed in those from zone III (Fig 2B). This differential expression contrasts with shaved roots, where *PvTET1A* transcript accumulation increases gradually beginning from zone I to zone II and from zone II to zone III (Fig 2A). Furthermore, an analysis of the reported expression atlas of *P*. *vulgaris* [55] indicates that *PvTET3* is ubiquitously expressed during the different root developmental stages, while *PvTET1A* is constitutively expressed in all root tissue, but with higher expression in some stages of root development (S5 Fig).

### *PvTET1A*, *PvTET8*, *PvTET3* and *PvTET4* are induced in response to NFs or rhizobia inoculation

We then evaluated the transcript accumulation of some tetraspanin genes in response to nanomolar concentration of NFs (10^−9^ M), which can reprogram polar growth and nodule primordia development [6]. In agreement with previous observations [6], treatment with 10^−9^ M NFs caused root hairs to swell and undergo morphological changes (S6B Fig). As expected, no morphological responses were observed when chitosan was used as a negative control (S6A Fig).

**Fig 3:**
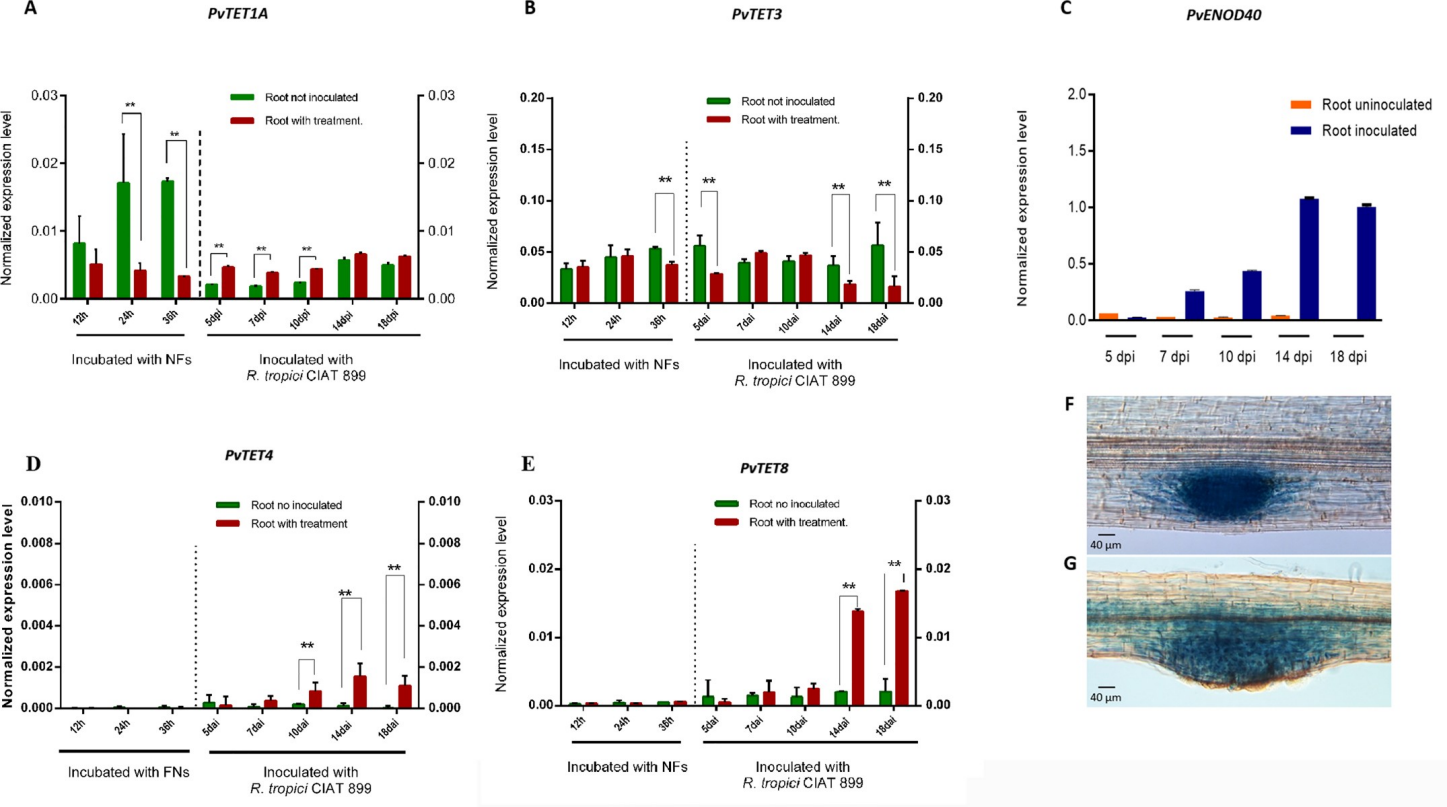
Expression of *PvTET1A*, *PvTET3*, *PvTET4*, and *PvTET8* in *P*. *vulgaris* during nodule development. Transcript accumulation is observed during nodule development under rhizobia colonization. (A) *PvTET1A*, (B) *PvTET3* and (C) *PvENOD40* transcript accumulation after Nod factor (NF) treatment or inoculation with *R*. *Tropici* CIAT 899 are depicted. *PvTET4* (D) and *PvTET8* (E) specifically respond to rhizobia inoculation. These results are compared with uninoculated roots harvested at the same time (green bars). Expression values were normalized with those of *EF1a*. Bars represent means ± SEM of at least three independent biological replicates with three technical repeats. P-values <0.05 are marked with two asterisks (Student’s t-test). Promotor activity of *PvTET8* in lateral root primordia (F) and nodule primordia development (G).

We initially considered to evaluate the transcript accumulation in response to NFs or rhizobia for *PvTET1A and PvTET3* since these genes were the most highly expressed in shaved root and root hairs under normal conditions, other tetraspanin genes were also tested, under NFs or Rhizobia inoculation, but the one that did not respond were not considered for further analysis (S7 Fig). *PvTET1A* was downregulated in *P*. *vulgaris* roots in the first 36 h days after inoculation with NFs, but we detected a response at 5–10 days post inoculation with rhizobia (Fig 3A). By contrast, *PvTET3* expression did not change within the first 24 h following inoculation with NFs, but a significative decrease was observed at 36 h after treatment. On the other hand, when inoculated with *R*. *tropici*, a further decrease was observed at 5 and 14–18 dpi (Fig 3B). Thus, some tetraspanin transcript levels respond to NF treatment or bacterial inoculation depending of the developmental stage, i.e at 5 dai, *PvTET1A* increases while *PvTET3* decreases. To confirm that *P*. *vulgaris* roots respond to rhizobia inoculation, we assessed the transcript accumulation of *PvENOD40*, an early nodulin gene induced during nodule development. *PvENOD40* is clearly induced after rhizobia inoculation, indicating that the nodule program is induced under our experimental conditions (Fig 3C).

**Fig 4:**
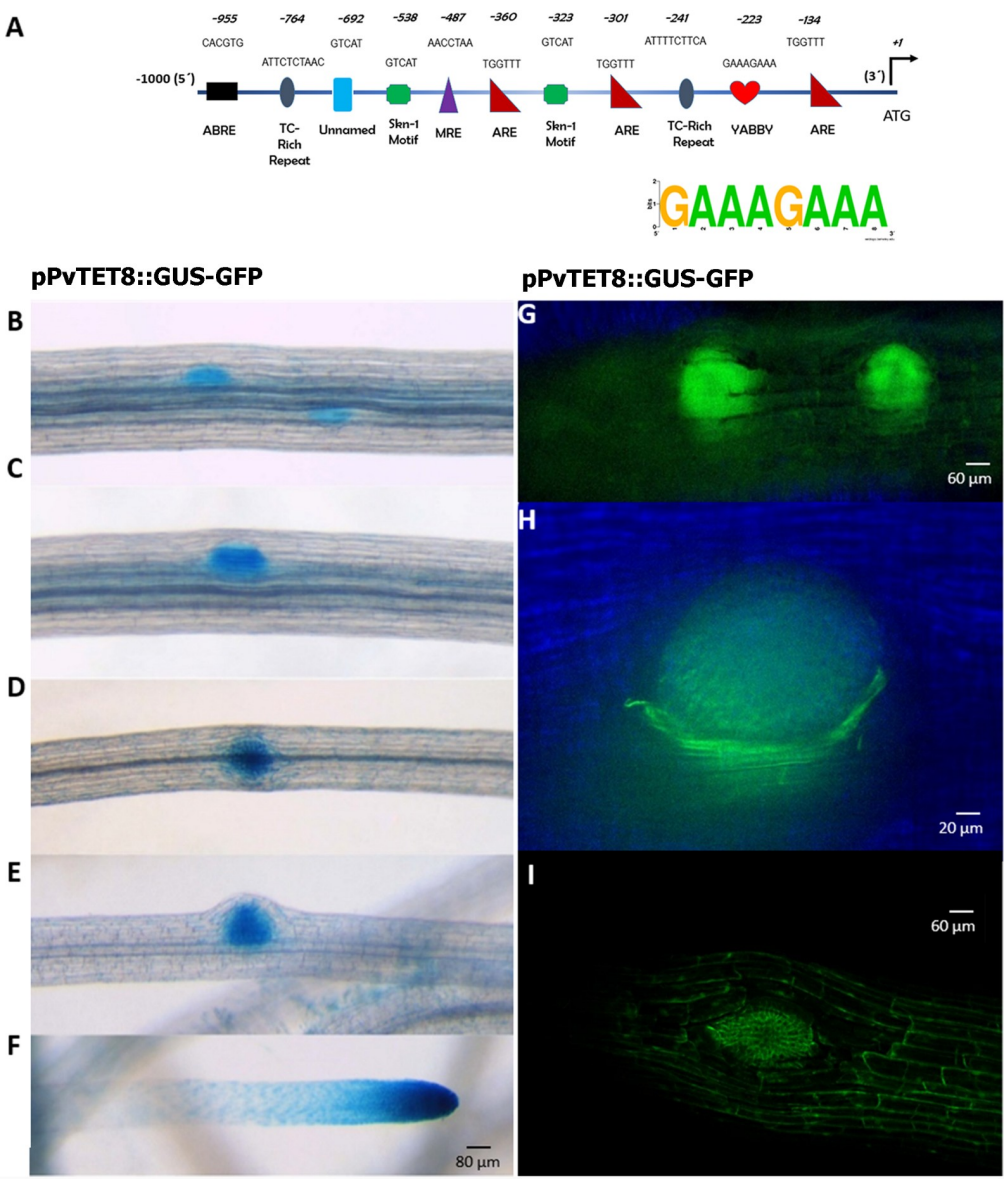
*PvTET8* transcript accumulation and promotor activity during lateral root and nodule primordia development. (A) Analysis of putative cis-regulatory elements in the promoter region of *PvTET8*. (B-F), *pPvTET8*::*GUS-GFP* promotor activity in *P*. *vulgaris* root during lateral root emergence. (G and H) show the promoter activity during the onset of lateral root development and the meristematic region of the emerging lateral root as depicted by fluorescence. (I), Subcellular localization of 35S:PvTET10-GFP during lateral root formation. Transgenic composite plants from *P*. *vulgaris* were generated by the *A*. *rhizogenes* method.

**Fig 5:**
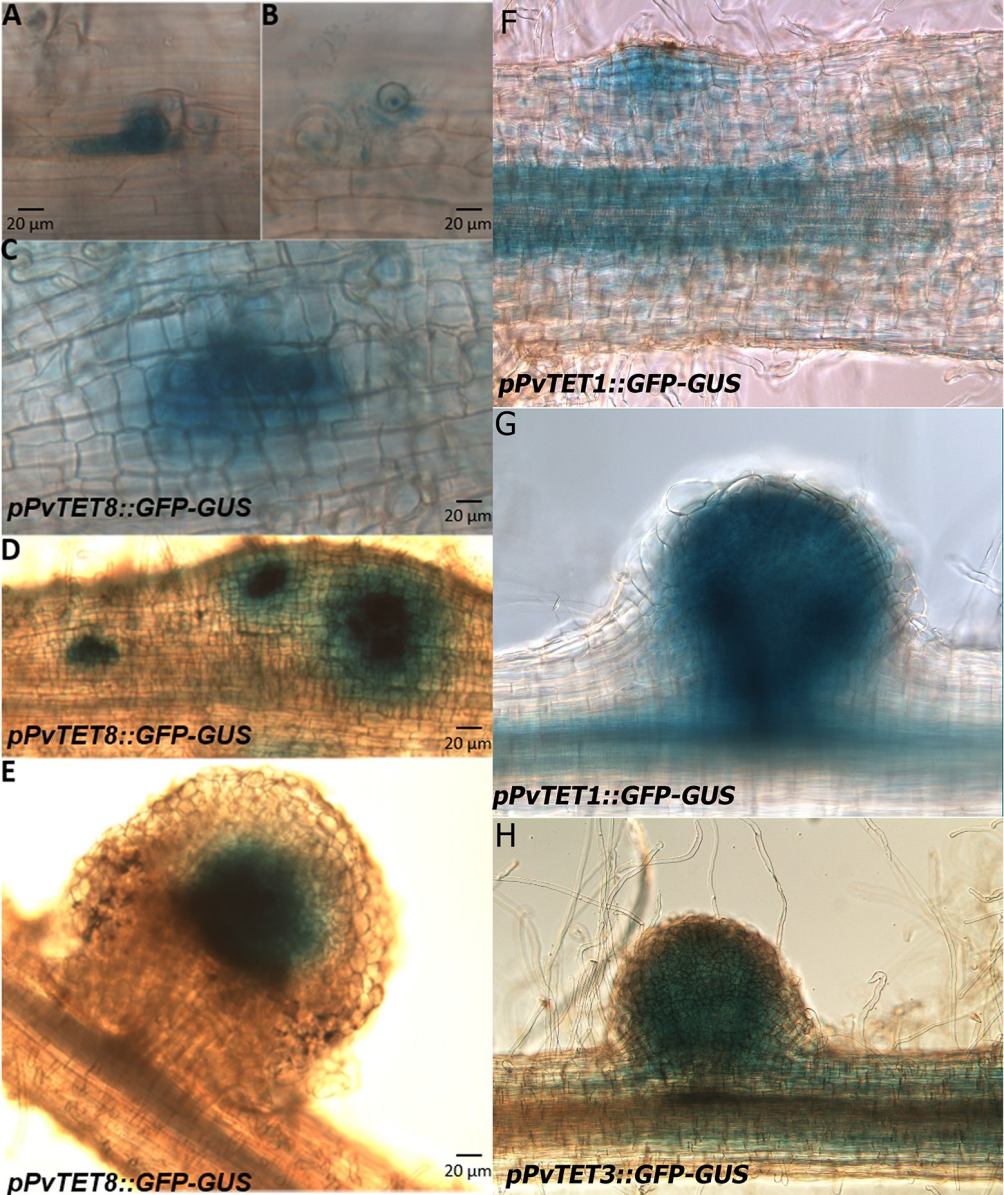
Promotor activity of *PvTET8* during nodule development and subcelular localization of PvTET6 in *P*. *vulgaris* during the infection process with rhizobia. (A-C) *pPvTET8*::*GUS-GFP* promotor activity at the early stages of infection thread formation in root hairs. (C and D) Promotor activity of *pPvTET8*::*GUS-GFP* during the early stages of cell division during primordia development and (E) in fully developed mature nodule, as depicted the promotor is highly expressed in the infection zone of the nodule. (F-G) Promotor activity for *pPvTET1A*::*GUS-GFP* and (H) promotor activity for *pPvTET3*::*GUS-GFP*. Transgenic composite plants were generated with *A*. *rhizogenes* and promotor expression analyzed by GUS activity. Bars represent 20 μm in all images.

We then further analyzed the transcript accumulation of *PvTET4* and *PvTET8* in roots treated with NFs and rhizobia. *PvTET4* was upregulated after *R*. *tropici* inoculation (Fig 3D), whereas *PvTET8* did not increase in response to NFs within the first 6 days, but it was specifically induced in response to rhizobia inoculation after 7 days (Fig 3E). Therefore, we selected *PvTET8* to examine whether transcript accumulation was correlated with promoter activity. We cloned the promoter and generated the *pPvTET8*::*GUS-GFP* construct, which revealed that *pPvTET8* was induced during nodule primordium development as well as in the meristematic region of the apical root, including the lateral root primordium (Fig 3F and 3G). We also observed clear promoter activity during the emergence of lateral root primordium, which originates from the pericycle (Fig 4B–4E), and this promoter activity remained in the root primordium (Fig 4F). Whereas the nodule primordium arises in the outer cells of the cortex (Figs 3G and 5D), the lateral root originates from the internal pericycle cells (Fig 3F). We examined promoter activity by assessing GUS activity (Fig 4B–4E) and fluorescence from the pPvTET8::GUS-GFP fusion. Both approaches yielded the same results (Fig 4G) including the pPvTET10::GUS-GFP (Fig 4I).

Next, we assessed *PvTET8* promoter activity during nodule development, from infection thread formation to fully grown nodules, and we found that the *pPvTET8*::*GUS-GFP* promoter is highly active during the early stages of nodule development, which includes infection thread formation in the root hairs (Fig 5A and 5B). Thereafter, when the cortical cells started to divide, forming cells that will give rise to the nodule primordium, clear promoter activity was observed in the outer cells that form the primordium (Fig 5C–5E). The fully developed nodule also depicts a clear and specific promoter activity in the infected zone of the nodule (Fig 5E). We also evaluated the promotor activity for *pPvTET1A*::*GUS-GFP* (Fig 5F and 5G) and *pPvTET3*::*GUS-GFP* (Fig 5H) the two most highly expressed gene in *P*. *vulgaris* root. Although these genes do not seem to have a strong increase during nodulation, when the promotor activity was assayed, there is a clear evidence that apart from the vascular bundle, the expression in the nodule is very clear.

Since *pPvTET8* was specifically induced in response to rhizobia inoculation, we analyzed the promoter sequence from *PvTET8* up to 1.0 kb upstream of the translation start site of *PvTET8* using PlantCARE (database) to identify putative *cis*-acting regulatory elements. In this analysis, we identified at least 14 different regulatory elements with a length of between 4 and 11 bp. Some *cis*-elements are represented in Fig 4A. The *cis*-regulatory elements TATA and CAAT were highly repeated in the promoter sequences. In addition, several putative *cis*-elements involved in the light, heat, and drought stress response, circadian control, defense, and anaerobic induction were present, as was one box associated with the YABBY transcription factor. Thus, *cis*-elements associated with defense and stress responsiveness and cell fate were well represented in this promoter region, as represented in Fig 4A.

**Fig 6:**
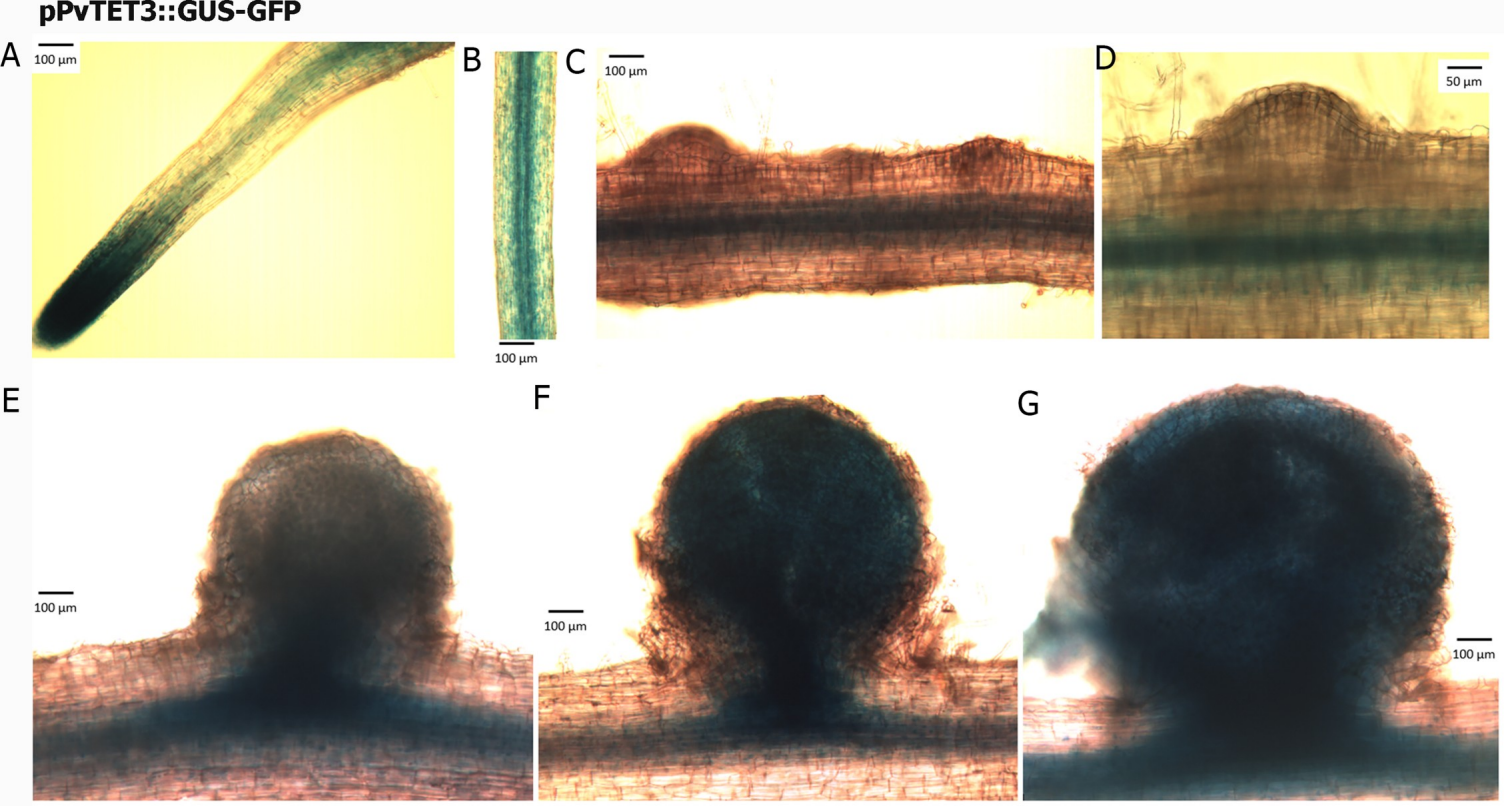
Promotor activity of *PvTET3* in the root and during nodule development in *P*. *vulgaris* during symbiotic conditions. (A and B) *pPvTET3*::*GUS-GFP* promotor activity in the apical root and vascular bundles. (C and D) *PvTET3* promotor activity at the early stages of nodule primordium formation. (E- G) Promotor activity during the nodule development. Transgenic composite plants were generated with *A*. *rhizogenes* and promotor expression analyzed by GUS activity.

Since *pPvTET3*::*GUS-GFP* was highly expressed in the root (Fig 5H), we prompted to explore if there is promotor activity during different stages of nodule development. We found that pPvTET*3*::*GUS-GFP* is expressed in the root meristematic region and vascular bundles (Fig 6A and 6B), including a low promotor activity during the early cell divisions in the nodule primordium (Fig 6C and 6D) and during the early nodule development (Fig 6E), again with higher activity in the vascular bundles, however, a clear localization in the nodule was found, although at latter stages of development in fully developed nodules (Fig 6F and 6G). Furthermore, the *pPvTET3* seems to be more localized in the cortex of the nodule, as compared to the *pPvTET8*, which seems more localized in the central region (Fig 5E) and most related to the infected region. These results suggest that both, *pPvTET8* and *pPvTET3* could be differentially expressed in the same organ, but in different region.

### Mycorrhizal association downregulates *PvTET3*, while phosphate scarcity upregulates *PvTET12* and downregulates *PvTET3*

**Fig 7:**
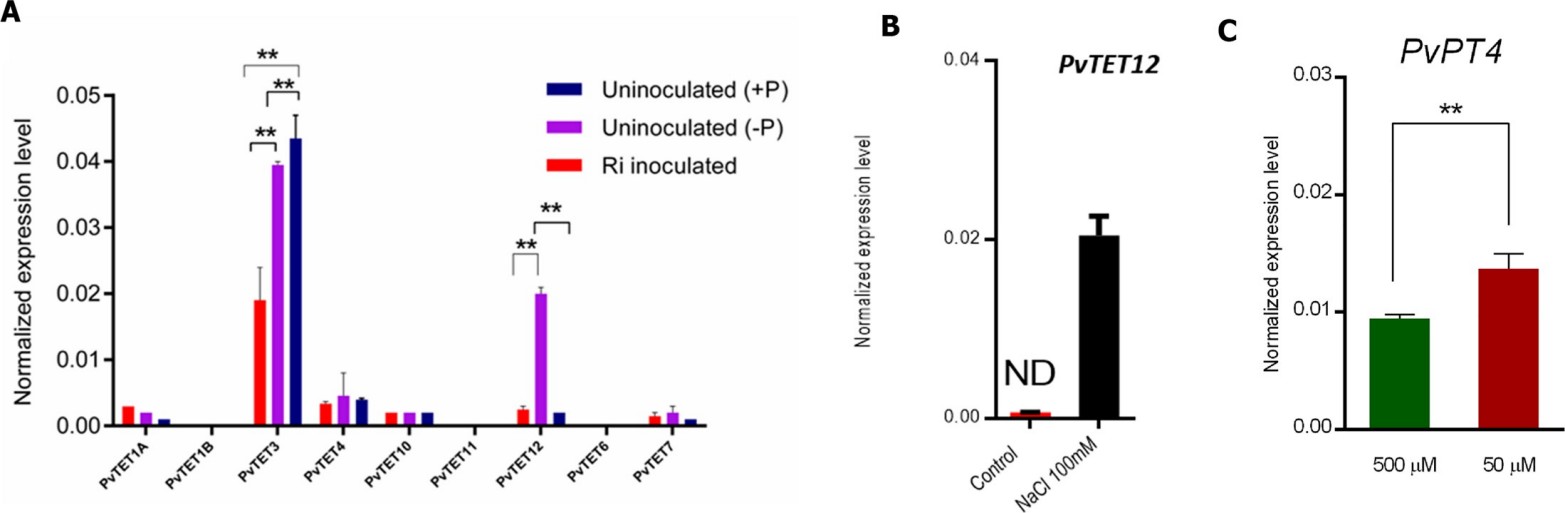
Tetraspanin transcript accumulation profile in *P*. *vulgaris* under *R*. *irregularis* colonization. (A) Tetraspanin transcript accumulation in *P*. *vulgaris* roots colonized with *R*. *irregularis* in the symbiotic stage at 6 wpi as compared with uninoculated plants under phosphate scarcity with potassium phosphate at 50 μM. Transcript accumulation was normalized to the expression of *Ef1a*, which was used as a reference gene. (B) Expression of tetraspanin *PvTET12* in *P*. *vulgaris* roots during abiotic stress induced by NaCl conditions at 100 mM at 24 hpi. (C) Phosphate transporter PT4 transcript accumulation under mycorrhizal condition. Data are the means ± SEM of two biological experiments (three roots collected from each biological experiment and for each period were used).

Since the nodulation process recruited many genes from the mycorrhizal association, we also explored if the tetraspanin genes could be modulated in response and mycorrhizal association and low phosphate. Therefore, we determined the effect of mycorrhizal interaction with *P*. *vulgaris* on the accumulation of different TET transcripts. We inoculated *P*. *vulgaris* seedlings with *R*. *irregularis* and included two controls, one in which plants were uninoculated and irrigated with a standard concentration of phosphate (500 μM) and another in which plants were uninoculated and irrigated with medium deficient in phosphate (phosphate scarcity at 50 μM), which is expected to induce the plant root response to phosphate scarcity, such as lateral root formation and root hair proliferation. Both controls were included to show the specific effect of mycorrhization on different tetraspanin transcript accumulation as a result of root hair proliferation and lateral root formation. While *PvTET8*, *PvTET4*, and *PvTET3* expression was modulated during nodule formation (Fig 3A and 3B and Fig 4A), inoculation with *R*. *irregularis* only affected *PvTET3* expression during the mycorrhizal association (see Fig 7A). As expected, the expression of the phosphate transporter PT4 which is induced during the mycorrhizal association, was found to be induced under our experimental condition (Fig 7C).

**Fig 8:**
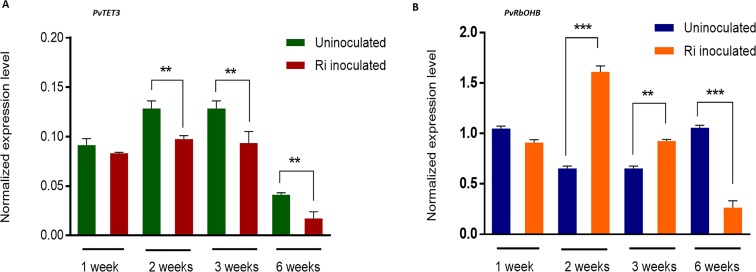
*PvTET3* and *PvRbohB* expression during mycorrhiza formation. **(A)** Quantitative RT-PCR analysis of relative expression levels of *PvTET3* in roots (wild type) inoculated with *R*. *irregularis* and under un-inoculated condition at the indicated number of weeks post-inoculation (wpi). Transcript accumulation was normalized to the expression of *Ef1a*, which was used as a reference gene. Data are the means ± SEM of two biological experiments (three roots collected from each biological experiment and for each period were used). (B) Expression of *PvRbohB* in *P*. *vulgaris* roots colonized by *R*. *irregularis*. Quantitative RT-PCR analysis of relative expression levels of *PvRbohB* in roots (wild type) inoculated with *R*. *irregularis* compared with expression in uninoculated roots of *P*. *vulgaris* at different weeks post-inoculation (wpi). Transcript accumulation was normalized to the expression of *Ef1a*, as a reference gene. The data are the means ± SEM of two biological experiments (three roots collected from each biological experiment and for each period).

A temporal analysis of *PvTET3* transcript accumulation under mycorrhization confirmed that *PvTET3* transcript levels were lower at 2, 3, and 6 weeks after mycorrhization compared with the control (Fig 8A). This suggests that *PvTET3* was downregulated during mycorrhization (Fig 7A and Fig 8A). It has been reported that transcript accumulation of *PvRbOHB* decreases during mycorrhizal association [56]. Therefore, we evaluated the accumulation of *PvRbOHB* transcript at 1, 2, 3, and 6 weeks post inoculation (wpi) to define its temporal response and correlate it with that of *PvTET3* (Fig 8B). *PvRbOHB* was not induced during the first week post inoculation, during which its expression was comparable to that of the control condition (Fig 8B). However, after 2–3 weeks, a clear increase in *PvRbOHB* transcript accumulation was observed, but a decreased *PvTET3* expression was found. However, at 6 weeks, a clear decrease in both *PvRbOHB* and *PvTET3* was observed, and thus confirming the previously reported data for *PvRbOHB* under mycorrhizal association [56].

We found no significant differences at the transcriptional level for the other tetraspanins under our experimental conditions (S7 Fig). However, we identified a clear *PvTET12* upregulation under phosphate scarcity (Fig 7A). It is interesting that under normal growth conditions, we did not observe significant *PvTET12* expression in root hairs at different developmental stages or even in the shaved roots (Fig 2A and 2B), even though its homolog, *AtTET12*, has been identified as a transcriptional signature in root hairs and pollen tubes [57]. To determine if another different stress, such as osmotic or saline treatment, could modify the *PvTET12* transcript level, we treated the *P*. *vulgaris* root with 100 mM NaCl; which is known to induce a saline response [58–60]. Under saline stress conditions, we found that *PvTET12* expression was also upregulated (Fig 7B).

### *P*. *vulgaris* tetraspanins localize to the apical plasma membrane, intracellular vesicles, and meristematic regions

**Fig 9:**
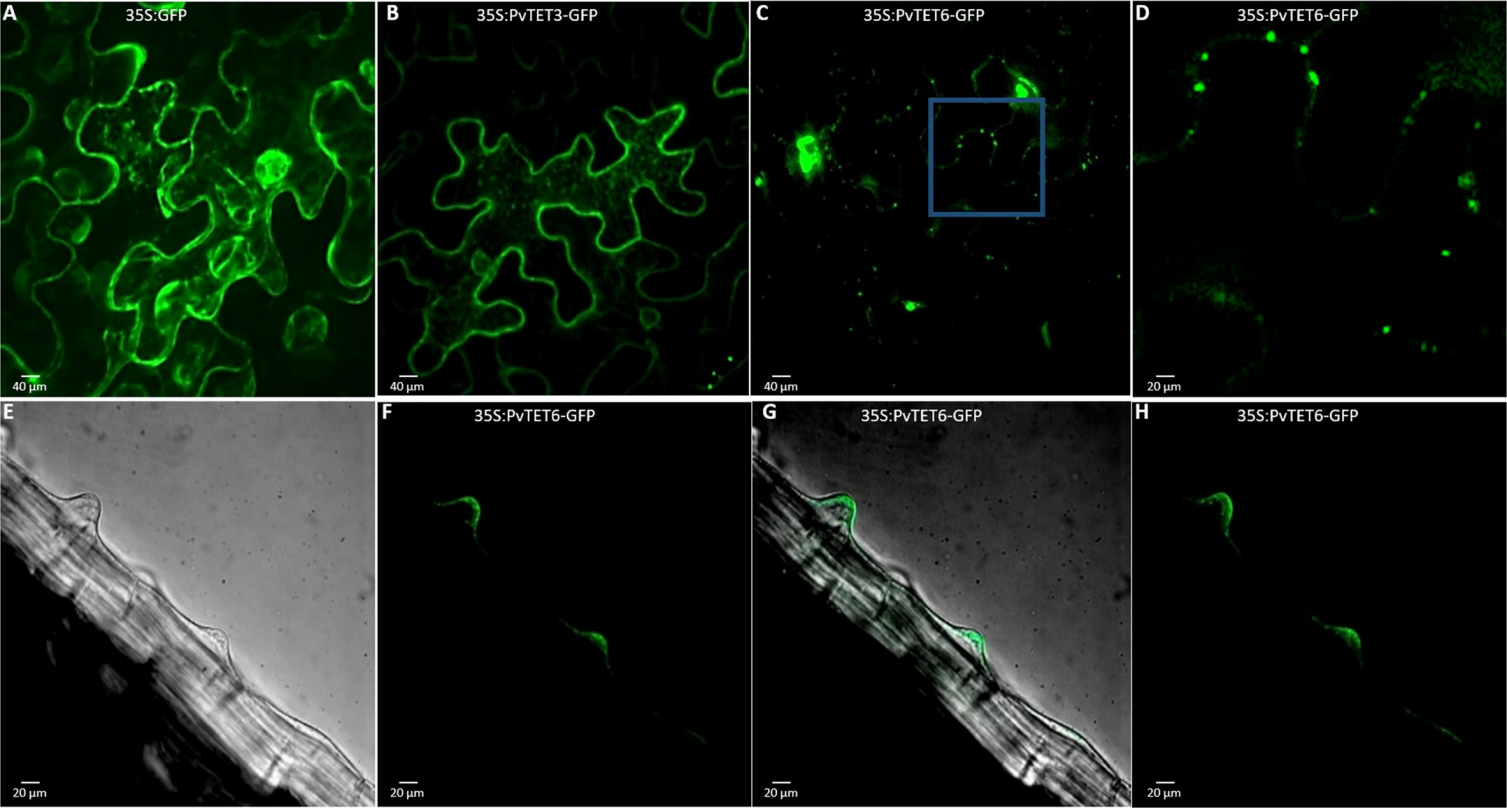
Subcellular localization of PvTET6 and PvTET3 in *Nicotiana benthamiana* leaves and growing root hairs of *P*. *vulgaris*. (A) Confocal analysis of GFP expression in the leaves of transgenic *N*. *benthamiana* plants. (B) 35S:PvTET3-GFP. (C) 35S:PvTET6-GFP. (D) 35S:PvTET6-GFP (close-up of region indicated in C). (E-H) 35S:PvTET6-GFP subcellular localization in transgenic living *P*. *vulgaris* root hairs. Images in bright field (E), Merge (G), GFP signal (F), Z-projection (H). Bars = 20 μm.

Tetraspanin proteins in general have been described to be plasma membrane proteins and component of cytoplasmic vesicles. However, TET3 in Arabidopsis has been found in proteomic analysis of plasmodesmata. Therefore, we determined the subcellular localization of *PvTET3* and *PvTET6* by generating GFP fusions of their encoding genes and transiently expressing these constructs in *N*. *benthamiana* leaves (Fig 9A–9D). An analysis of leaves expressing the 35S:PvTET3-GFP and 35S:PvTET6-GFP constructs under the control of a constitutive promoter (CaMV35S) showed that these proteins examined in this study accumulated at the periphery of *N*. *benthamiana* epidermal cells, indicating a membrane localization (Fig 9B and S8 Fig), and clearly differentiated from the cytoplasmic localization of GFP in control agroinfiltrated cells (Fig 9A). Furthermore, some of the tetraspanins localized to fluorescent spots in the plasma membrane, suggesting plasmodesmata localization (Fig 9C, inset). We also generated transgenic *P*. *vulgaris* composite plants expressing 35S:PvTET6-GFP using *A*. *rhizogenes* and found that some tetraspanins are expressed in vesicular structures that are swept along by cytoplasmic flux in growing root hairs, but are also localized in the apical plasma membrane of these cells (Fig 9E–9H, S1 Movie and S2 Movie). In order to determine the plasma membrane localization, we generated agroinfiltrated *N*. *benthamiana* epidermal cells expressing the tetraspanin and subjected to NaCl treatment for plasmolysis. These results suggest that the signal remains associated to the plasma membrane (S8 Fig).

**Fig 10:**
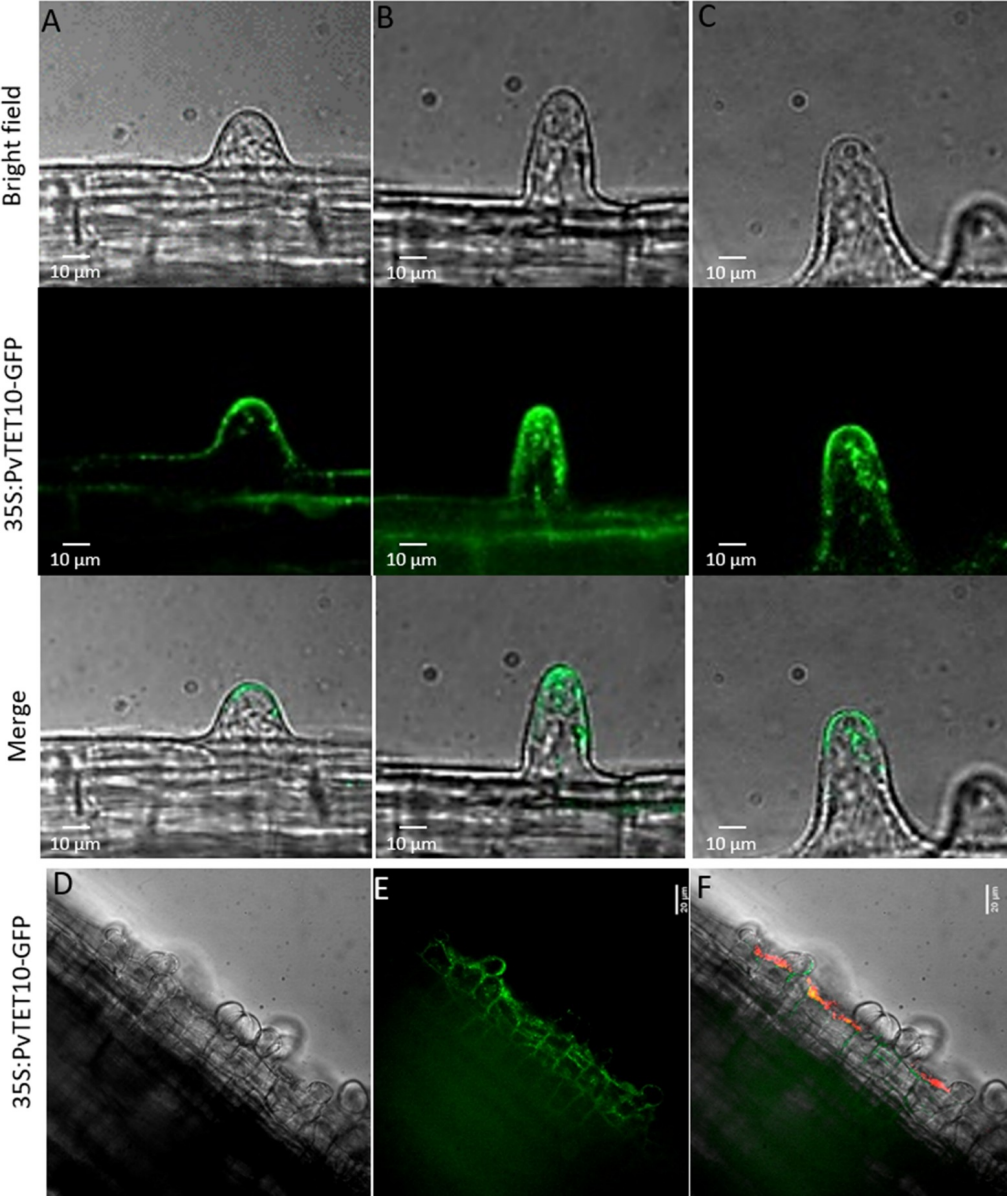
Subcellular localization of PVTET10 in growing root hairs from *P*. *vulgaris*. (A, B and C) Apical membrane localization of 35S:PvTET10-GFP at different developmental stages of the growing root hair. (D, E and F) Cytoplasmic vesicle localization for PvTET10, and its accumulation in the infection site where the infection thread and nodule primordia are induced. *P*. *vulgaris* plants were transformed by *A*. *rhizogenes* in order to generate the composite plants. Bacterial colonization is in red and the subcellular localization of PvTET10 is in green.

We then evaluated 35S:PvTET10-GFP expression in composite plants. TET10 exhibited similar localization to TET6, namely, apical localization in the plasma membrane and dynamic vesicles in the cytoplasm of growing root hair cells in a pattern that sometimes follows the cytoplasmic streaming with higher ambulation at the tip dome (Fig 10A–10C). Furthermore, in *P*. *vulgaris* composite plants expressing 35S:PvTET10-GFP under nodulation conditions, the cortical cells that enter the division process that will generate the nodule primordium are enriched in these cytoplasmic vesicles (Fig 10D–10F). Since TET3, TET6, and TET10 localized to moving vesicles, we further examined the dynamics and behavior of these vesicles.

**Fig 11:**
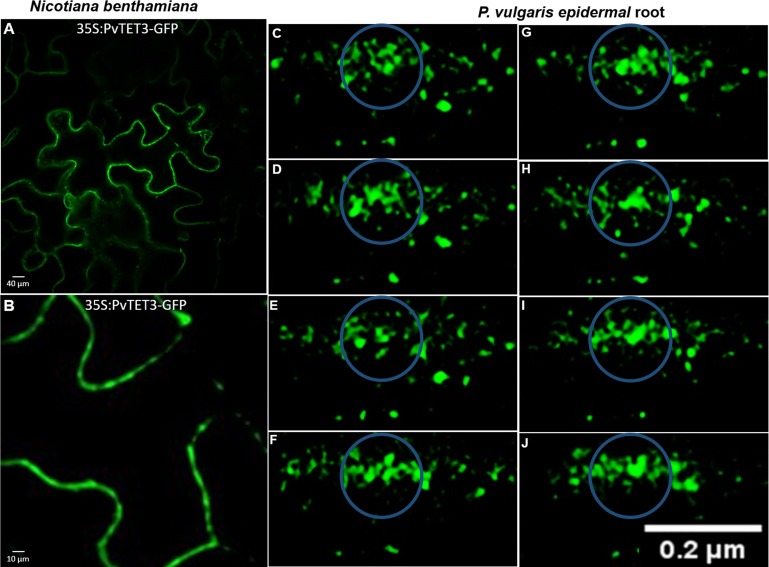
Subcellular localization of PvTET3 in *N*. *benthamiana* leaves and *P*. *vulgaris* roots. (A and B) 35S:PvTET3-GFP localization in cytoplasmic vesicles in agroinfiltrated *N*. *benthamiana* leaves. (C-J) Subcellular localization of 35S:PvTET3:GFP in *P*. *vulgaris* composite epidermal root cells. Cytoplasmic vesicles were tracked over time in order to show the fusion and morphological changes resembling protrusions during the cytoplasmic streaming. Vesicles were tracked in a time lapse of 1.6 seconds for 3 min in transgenic roots expressing 35S:PvTET3-GFP.

We selected PvTET3, which we know localizes to vesicular structures in *N*. *benthamiana* (Fig 11A and 11B) and *P*. *vulgaris* composite plants (Fig 11C–11J), to determine the dynamics and shapes of these vesicles. In *P*. *vulgaris* epidermal cells, these vesicles were tracked in a time lapse of 1.6 seconds for 3 min in transgenic roots overexpressing 35S:PvTET3-GFP. We observed fusion events that resulted in larger vesicles, with amorphous shapes that resemble protrusions (Fig 11C–11J).

## Discussion

We found that *PvTET1A*, *PvTET3*, *PvTET4* and *PvTET8* are induced and differentially expressed during early nodule development, as depicted by transcript accumulation and promoter activity and in the fully grown and mature nodule, but with different timing and strength. Furthermore, these expression patterns resemble those found in primordia during lateral root formation which also requires a new program that involves cell division and ROS production. The finding that some tetraspanin presents a clear membrane localization at the tip of the root hairs and some others in what appears to be associated with plasmodesmata (PD), and cytoplasmic vesicles, suggest a role in symplastic communication trough regulation of PD or in cell trafficking as described in animal cells [18].

We identified the tetraspanin family in *P*. *vulgaris* and found *PvTET10* to be a conserved tetraspanin with at least one representative in each legume species examined, except for soybean, which contained more members due to a genome duplication event [61]. *PvTET10* clusters with *AtTET10* and *OsTET14*, which contain similar structural features of introns/exons, suggesting that *PvTET10* is the ancestral tetraspanin gene, as is AtTET10 in the Brassicaceae and *OsTET14* in *O*. *sativa* [35, 50]. These structural intron/exon patterns indicate a similar origin as suggested for deuterostomes, in which tetraspanins are the product of a divergent evolutionary process and originated from at least one common ancestor [51, 62]. In our unrooted phylogenetic tree, *PvTET1A* and *PvTET1B* grouped with *AtTET1* (*TORNADO*) and *AtTET2*, while *PvTET8*, *OsTET7-9*, and *AtTET8-9* clustered together (S2 Fig and S3 Fig).

We found that *PvTET3* and *PvTET1A* transcripts are more abundant and ubiquitously expressed during root and root hair development (Fig 2A and 2B). In angiosperms plants such as *Arabidopsis* and *P*. *vulgaris*, primary root formation takes place during embryogenesis, whereas lateral roots form post-embryonically from pericycle founder cells, and both of these processes are mediated by changes in hormone levels [63, 64]. *PvTET1A* is expressed post-embryonically, between 2 and 4 dpg, in the seedling when root growth begins and before lateral roots emerge [38]. According with our phylogenetic data, *PvTET1A* is the homolog of *TORNADO* in *Arabidopsis* (*AtTET1* or *AtTRN1*), which regulates cell specification in the root epidermis during radial pattern formation and thus in agreement with our transcriptional profile for *PvTET1A* with a high expression in root [65]. However, our transcriptional profile does not correspond with the reported higher *TRN1* promoter activity in the meristematic zone in the root tip. This discrepancy with higher expression in root zone II and III and lower in zone I that correspond to the meristematic region could be due to the fact that the promoter region of *PvTET1A* does not contain a putative cis-regulatory element, GCCACT, that exists in *Arabidopsis TRN1*, and associated with meristematic expression and auxin dependency [65, 66]. Therefore, *PvTET1A* could have a different regulation, or its expression pattern could differ from that reported in *Arabidopsis*.

The finding that *PvTET3* transcript accumulation was downregulated under nodulation and mycorrhizal conditions in *P*. *vulgaris* roots and that the encoded protein localizes to the PM and PD, suggests that PD could be a regulated structure during the mutualistic interactions. We also found that *PvTET6* is specifically targeted to the PM and PD, in agreement with the subcellular localization of some homologous tetraspanins identified in *Arabidopsis*, such as *AtTET3* and *AtTET5* [35, 67–71]. PD are connections between cells that mediate symplastic communication from single cells to tissue domains, these structures have essential roles in cell-to-cell communication [72]. Both the density and aperture size of PD are developmentally regulated through the deposition or solubilization of callose by callose synthases or glucanases, allowing the formation of spatial symplastic domains that establish tissue-specific developmental programs [73]. Numerous non-cell-autonomous proteins (NCAPs) and small RNAs travel through the PD and play crucial roles in cell fate determination and organ patterning during plant development. In vascular plants, it has been suggested that PD networks are associated with shoot apical meristem (SAM) organization in *Arabidopsis* and maize (*Zea mays*) [74–76]. Typical PD proteins, such as AtPDLP1, have a specific signal peptide, LVL, located in a transmembrane domain [67], which was also found in PvTET6 and PvTET3. Transmembrane domains in tetraspanin have turned to play important roles. For instance, a single point mutation, L31S substitution in the third amino acid of TM1 has been associated with field-evolved resistance of cotton bollworm to transgenic Bt cotton [77].

In rice, blast disease caused by the hemibiotrophic fungus *Magnaporthe oryzae* grow from one cell to the next through PD. This response is coordinated by chitin perception that requires a receptor kinase (CERK1) and a chitin elicitor binding protein (CEBiP), which sense the chitin and induce a reduction in the cell-to-cell connectivity via PD [80, 81]. During the nodulation process it has been recently described that PD connection between the phloem-early primordium-epidermal cell forming the infection thread is a key step for the infection process [82, 83]. Again, it seems that PD are key components of the photogenic and mutualistic response and PD localized tetraspanins could be important modulators of the intercellular flux regulating the symplastic continuity and molecular flux between cells. It could involve ROS to regulate PD permeability by facilitating the cross-linking process of callose [84]. Therefore, the observed downregulation or upregulation of *PvTET3* could affect PD composition by modulating the capability of tetraspanin to recruit some additional molecular components required to modify the callose composition in PD during mycorrhizal or rhizobia association. This may also facilitate the hypha or infection thread to move cell-to-cell through the PD connections or facilitate the diffusion of some proteins, such as transcription factors or metabolites, required to fine-tune the regulation of cortical and cortex responses.

Nodulation involves substantial crosstalk between NFs and auxin signaling in *Medicago truncatula*, with a high accumulation of auxin at the site of nodule meristem formation [85]. Indeed, there is a large overlap between genes induced in response to NFs and auxin, including two tetraspanin genes with homology to *PvTET3* (Medtr4g061010) and *PvTET1A* (Medtr8g101600) [85]. In addition, a previous report showed that *PIN1* expression was reduced in the *Arabidopsis trn2-1* mutant, which has compromised auxin transport activity during the transition from floral meristem termination to gynoecium development, which suggests a link between tetraspanin and auxin homeostasis [86]. Therefore, in addition to altering auxin transport, Nod factors could modulate the expression of specific tetraspanins that influence hormone levels, which could regulate cell division, a well-described process during nodulation [87]. If *PvTET1A* and *PvTET3* are downregulated during the interaction with NFs, this response could be associated with the disruption of auxin transport or the stimulation of auxin biosynthesis [88]. We suggest that NFs signaling has a profound impact on *PvTET1A* and *PvTET3* expression by affecting auxin levels and thereby coordinates nodule primordium development. Indeed, it is well known that NFs interfere with auxin transport, biosynthesis, and homeostasis [87, 89].

The finding that *PvTET4* and *PvTET8* are induced during early nodule development with different timing and localization, as depicted by transcript accumulation and promoter activity and in the fully grown and mature nodule. Our results indicate that *PvTET8* could be involved at different developmental stages of nodule primordia formation and in the infected thread region, and *PvTET3* although we did not find a high transcript accumulation, the promotor activity indicates that is expressed in root and mature nodule, suggesting a role in the mature nodule or later on during senescence. Furthermore, these expression patterns resemble those found during lateral root formation. Both lateral root primordium and nodule primordium formation require differentiated cells to become dedifferentiated and then to enter a new program that involves cell division. Therefore, this tetraspanin could be involved in the molecular mechanism that maintains meristem activity.

Furthermore, the roles of tetraspanin have been expanded beyond intercellular boundaries. It has been reported recently that *Arabidopsis* cells secrete exosome-like extracellular vesicles that are derived from the multivesicular bodies (MVBs), which contain cytoplasmic material, such as proteins, miRNA, and lipids [23, 90]. Under certain circumstances, these MVBs fuse to the plasma membrane and release the internal vesicles into the extracellular space. It has been demonstrated recently that tetraspanins are key components and specific markers for animal (CD63) and plant exosomes (*AtTET8* and *AtTET9*) [23]. In *Arabidopsis*, exosomes transport key miRNAs that induce silencing of fungal genes critical for pathogenicity in *Botrytis cinerea*, defining a role for tetraspanin and exosome biogenesis in intercellular and inter-kingdom communication [23]. This could explain why *AtTET8* expression is upregulated upon treatment with pathogen elicitors [38] and why, during our experimental conditions, *PvTET8* is induced when plants are infected with rhizobia. It is possible that exosomes could play an important role during the mutualistic interaction. Furthermore, tetraspanin genes are among the induced genes in the reported legume transcriptome of wild peanut (*Arachis spp*) roots infected with nematodes, suggesting that the pathogenic response in legumes also involves tetraspanins [91].

The upregulation of *PvTET8* during the defense response also can be explained by the enrichment of defense response cis-regulatory elements in its promoter region. Indeed, it has been widely suggested that the molecular mechanism underlying mutualism was derived from that underlying pathogenic interactions, but suppression of the immune response allows the symbiont to colonize the plant host [92]. It will be interesting to determine the specific role of *PvTET8* during nodulation and mycorrhization and explore the role that vesicles and exosomes could play in recruiting proteins or membrane-associated proteins that are required in the plasma membrane during mutualistic interactions.

Our data showing that *PvTET12* is upregulated under phosphate scarcity, but not under mycorrhizal association, suggest that *PvTET12* expression is highly dependent on environmental nutrition conditions. In *Arabidopsis*, *AtTET12* has been identified in root hairs and in the pollen tube [57]. Furthermore, the microarray data of *Glycine max* reported on the Soybean Efb Browser website shows a differential expression of *GmTET12* in roots and root hairs at 24 h after inoculation with bacteria [35, 57, 93]. These data suggest a role for *PvTET12* in the massive proliferation of root hairs as a response to phosphate acquisition [94–96].

In animal cells, tetraspanins have multiple antagonistic effects. For example, the tetraspanin CD82 is downregulated during metastases, while the tetraspanin CD151 and tetraspanin 8 are induced and able to support tumor progression [21, 22]. It has been reported that the CD63 tetraspanin in animal cells recruits a H^+^-ATPase beta-subunit in parietal cells that affects its trafficking. Also, saline stress has been used to induce changes in the H^+^-ATPase localization mediated by tetraspanin [97, 98]. Here, we also found that NaCl could upregulate *PvTET12*. Furthermore, in *Arabidopsis*, a mutant of one isoform of PM H^+^-ATPase, *AHA7*, exhibited reduced root hair density and lower H^+^ density efflux in the root hair zone, while the transcript was upregulated under low-phosphate conditions [99]. Since phosphate scarcity affects *PvTET12* expression, it could affect the recruitment of protein related to the change in H^+^ efflux, and it is tempting to think that tetraspanins are somehow related to the regulation or trafficking of H^+^-ATPase. Furthermore, a PT4/PT11 H^+^-ATPase has been found to be important for arbuscule maintenance and AM-mediated phosphate uptake [100, 101]. Plants have developed several strategies to increase phosphate acquisition, including changes to root architecture and the formation of root hairs [95, 102]. However, the finding that other TETs, such as tetraspanin-1, are also related to changes in root hair regulation suggests the existence of a more complex regulation [65].

There is an emerging association between tetraspanins and the mechanisms of ROS generation [24]. In the nematode *C*. *elegans*, the exoskeleton, the cuticle composed of collagen, is tyrosine cross-linked in a ROS-dependent manner, in a process assisted by BLI-3, a DUOX NADPH oxidase [29]. This process also requires the participation of tetraspanin TSP-15, which allows recruitment of the NADPH oxidase [24, 25, 30]. Inactivation of this tetraspanin or BLI-3 produces similar phenotypes. Furthermore, during plant infection with the pathogenic fungus *M*. *grisea* or *B*. *cinerea*, the tetraspanin PLS1 is required for infection site formation. Therefore, the tetraspanin and ROS generated by a NADPH oxidase is needed to coordinate ROS production at the infection site. In pathogenic *C*. *lindemuthianum*, a tetraspanin is also required to reestablish appressorium polarity [24, 25, 31–33]. Furthermore, in *Claviceps purpurea*, Nox2 and Pls1 are important for a balanced host–pathogen interaction, while in HeLa cells, the cotransfection of tetraspanin CD82 and GTPase Cdc42 induces apoptosis by generating ROS [103, 104]. These data add weight to the strong connection between tetraspanins and the ROS-generating machinery, both in animal and plant cells, to coordinate localized ROS production [24, 25, 30, 105].

During the mycorrhizal association of *P*. *vulgaris* with *R*. *irregularis*, only *PvTET3* was downregulated. This response coincides with previous reports in which a decrease in ROS production induced by RNAi of *PvRbOHB* enhanced colonization by *R*. *irregularis* [56]. Thus, a coordinated decrease in ROS production and *PvTET3* expression could be required for mycorrhizal colonization, as previously suggested [56, 78, 79].

This spatial ROS requirement is also observed during the Casparian strip lignification that occurs in plants, where CASP, a protein recruiting the NADPH oxidase, plays a role similar to tetraspanin, bringing together NADPH oxidase and peroxidase and ensuring localized activation of the oxidase [106]. Tetraspanins have also been localized to the tip of growing pollen tubes and root hairs (this work), and it is well known that the tip regions require NADPH oxidase activity for the generation of ROS, key players in the regulation of polar growth [12, 13]. The subcellular localization of some tetraspanins at the apical plasma membrane suggests that tetraspanins may play a key role during polar growth. Furthermore, the particular expression of *PvTET8* in root hairs in which an infection thread forms during early cell division in cortical cells could be related to the NADPH-oxidase-mediated ROS generation that is required for meristematic activity. Therefore, it is tempting to speculate that tetraspanins also play a role in recruiting the ROS-generating machinery to a specific cellular location. Moreover, tetraspanin also accumulates at the site of female gametophyte differentiation, a well-described ROS-dependent process [35].

Finally, the site-specific localization of *PvTET8* in root and nodule meristematic regions points to a role in meristem maintenance. The well-described role of O_2_^.-^ accumulation in the root apical meristem (RAM) and shoot apical meristem (SAM) in inhibiting the transition from proliferation to differentiation suggests a key role for tetraspanins in undifferentiated cells to inhibit differentiation in plant. Indeed, the balance of O_2_^.^/H_2_O_2_ levels in undifferentiated and differentiated cells is crucial for WUSCHEL (WUS) activation to promote stem cell differentiation. For instance, ROS can activate *WUS* and thereby repress expression of the TF YABBY, which regulates the tetraspanin *TORNADO* at the transcriptional level. Therefore, the tetraspanin *TORNADO* could be involved in processes that regulate ROS production, both at the temporal and spatial level. It is interesting that the WUS-RELATED HOMEOBOX (*WOX*) family transcription factor *WOX5* like *PvTET8*, *PvTET3* (this work), are also highly expressed during nodule organogenesis, suggesting that *WOX* genes are common regulators of cell proliferation in different systems, such as the SAM, RAM, and nodule primordium, including in megasporogenesis [36, 40, 86, 107].

Calcium and ROS are important cellular messengers and key players during mutualistic interactions [9, 108]. ROS, and therefore the enzymes that generate ROS (e.g., NADPH oxidases), play a key role in root hair tip growth, both in the presence and absence of pathogenic or mutualistic interactions, and in the growth of other tip-growing cells such as pollen tubes [12]. The observation that FNs from rhizobia have a different effect on ROS accumulation than do pathogenic signals, such as elicitors, suggests that plant cells differentiate symbiotic from pathogenic signals [6, 14, 109]. We previously reported that *P*. *vulgaris* produces 9 NADPH oxidases (Rboh), some of which are mainly expressed in roots, root hairs, or nodules [43]. The overexpression of one, *RbohB*, results in increased nodulation, but with a reduced mycorrhizal association [56], suggesting that ROS are important players in mutualistic interactions [42, 43, 56]. We suggest that the co-occurrence of tetraspanin and NADPH oxidase in the apical root hair cells or the early infection thread, and nodule primordia, could be related to the ROS-generating machinery. The role of tetraspanins in regulating the plasma membrane by recruiting the required proteins to specific membrane microdomains enriched in tetraspanins (tetraspanin web) or by affecting its vesicular trafficking have been well described. It is important to bear in mind that the release of the rhizobia from the infection thread or the arbuscule formation requires a complete coordination with the secretory system from the host plant cells for symbiont accommodation. This involves a high rate of exocytosis of specific components required for cell wall remodeling, including the extra membrane required to form the peribacteroideal membrane. In this scenery, tetraspanins could also play a central role organizing those membrane domains, but also facilitating the required vesicular trafficking to specific places as those reported for photogenic interactions that involves the specific exosomes secretion.

## Supporting information

S1 Fig Oligonucleotides used in this study. (DOCX)

S2 Fig Phylogenetic tree of TET family proteins from *Arabidopsis* and *P*. *vulgaris*. The phylogenetic tree was generated from the alignment of tetraspanin proteins with *n* = 1000 bootstrap replicates. The TET proteins were classified into clades based on phylogenetic analysis using the neighbor-joining (NJ) method. We used as query all tetraspanins reported by Boavida et al., 2013.
(TIF)

S3 Fig An unrooted neighbor-joining three phylogenetic tree was constructed based on the amino acid sequences alignment of some legumes tetraspanins using the neighbor-joining method (NJ)(Saitou and Nei, 1987, Takata et al., 2013). We selected amino acid sequences from *Medicago truncatula*, *Phaseolus vulgaris*, *Glycine max*, and *Lotus japonicus*. In this phylogenetic tree we schematize seven groups formed with legumes tetraspanin and are represented by different color branch. We selected a bootstrapping method to build the phylogenetic tree with 1000 replicates using MEGA Version 6.0.6 (Tamura et al., 2013)
(TIF)

S4 Fig Rab2A transcript levels during root hair development. Transcript levels were quantified by reverse transcription and real-time PCR (RT-qPCR) and calculated using the expression levels of *Elongation Factor 1α* as reference. Measures were performed in each enriched tissues and different zones in root of common bean at 48 hpg. The number of biological replicates (*n = 3*) is indicated. Error bars indicate mean and SEM (±SEM).
(TIF)

S5 Fig Expression level reported in transcriptomic atlas of common bean. **FY**- Young flowers, collected prior to floral emergence; **LF**- Leaf tissue from fertilized plants collected at the same time of LE and LI; **L5**- Leaf tissue collected 5 days after plants were inoculated with effective rhizobium; **LE**- Leaf tissue collected 21 days after plants were inoculated with effective rhizobium; **LI**- Leaf tissue collected 21 days after plants were inoculated with ineffective rhizobium; **N5**- Pre-fixing (effective) nodules collected 5 days after inoculation; **NE-** Effectively fixing nodules collected 21 days after inoculation; **NI**- Ineffectively fixing nodules collected 21 days after inoculation; **P1**- Pods between 10 and 11 cm long, associated with stage 1 seeds (pod only); **P2**- Pods between 12 and 13 cm long associated with stage 2 seeds (pod only); **PH**- Pods approximately 9cm long, associated with seeds at heart stage (pod only); **PY-** Young pods, collected 1 to 4 days after floral senescence. Samples contain developing embryos at globular stage; **R**- Whole roots from fertilized plants collected at the same time as RE and RI; **R5**- Whole roots separated from 5 day old pre-fixing nodules; **RE**- Whole roots separated from fix+ nodules collected 21 days after inoculation; **RI**- Whole roots separated from fix- nodules collected 21 days after inoculation; **RT**- Root tips, 0.5 cm of tissue, collected from fertilized plants at 2nd trifoliate stage of development.; **S1**- Stage 1 seeds, between 6 and 7 mm across and approximately 50 mg; **S2**- Stage 2 seeds, between 8 and 10 mm across and between 140 and 150 mg; **SH**- Heart stage seeds, between 3 and 4 mm across and approximately 7 mg; **ST**- Shoot tip, including the apical meristem, collected at the 2nd trifoliate stage; **YL**- Fully expanded 2nd trifoliate leaf tissue from plants provided with fertilizer; **YR**- Whole roots, including root tips, collected at the 2nd trifoliate stage of development; **YS**- All stem internodes above the cotyledon collected at the 2nd trifoliate stage. Common bean atlas source (https://plantgrn.noble.org/PvGEA/blastprotein.jsp).
(TIF)

S6 Fig Common bean root responses to NFs treatment. **(A)** Representative image of root hairs of roots of common bean under control condition treated with chitosan 10^−9^ M and **(B)** root hair subjected to a treatment with 10^−9^ M of NFs for 4 h (Scale = 100 μm).
(TIF)

S7 Fig Expression of the other of tetraspanin members in *P*. *vulgaris* during nodule formation. Different transcript abundance under two treatments: incubated with Nod Factor or inoculated with *R*. *Tropici* CIAT 899 (separated with a dotted line). The experiment included uninoculated roots harvested as control at the same time (green bars). Expression values were normalized with EF1a. Bars represent means ± SEM from at least three independent biological replicates with three technical repeats. P-values <0.05 are marked with two asterisks, respectively (Student’s t-test).
(TIF)

S8 Fig Subcellular localization of 35S:PvTET3-GFP, 35S:PvTET6-GFP after plasmolysis. Agroinfiltrated cells from *N*. *benthamiana* leaves under plasmolysis induced by NaCl. **A**, **B** and **C**, 35S:GFP, 35S:PvTET3-GFP, 35S:PvTET6-GFP respectively, showing the regular cytoplasmic protein localization under control condition (left panel) and under plasmolysis (right small panels). Arrows in **B** and **C** indicates the 35S:PvTET3-GFP and 35S:PvTET6-GFP fluorescence associated with the retracted plasma membrane, while the 35S:GFP remains in the cytoplasm in **A**.
(TIF)

S1 Movie Composite plants from *P*. *vulgaris* expressing the fusion protein 35S:PvTET6-GFP. Images were acquired with a spinning disk confocal system (Intelligent Imaging Innovations /3i, USA) consisting of a CSU-W1 confocal head (Yokogawa, Japan) and a modular solid-state laser stack; Slidebook software was used to control the system and capture images (Intelligent Imaging Innovations /3i, USA). Movie represent 40 images taken 15 seconds apart. Note the apical membrane localization and the cytoplasmic localization in vesicular structures that follows the cytoplasmic streaming.
(WMV)

S2 Movie Composite plants from *P*. *vulgaris* expressing the fusion protein 35S:PvTET6-GFP in epidermal cells. Images were acquired with a spinning disk confocal system (Intelligent Imaging Innovations /3i, USA) consisting of a CSU-W1 confocal head (Yokogawa, Japan) and a modular solid-state laser stack; Slidebook software was used to control the system and capture images (Intelligent Imaging Innovations /3i, USA). Movie represent 40 images taken 15 seconds apart. Note the apical membrane localization and the cytoplasmic localization in vesicular structures that follows the cytoplasmic streaming.
(AVI)
