# Supplementary material for: Differential tetraspanin genes expression and subcellular localization during mutualistic interactions in Phaseolus vulgaris
Source: PLoS One. 2019 Aug 22;14(8):e0219765. doi: 10.1371/journal.pone.0219765 (PMC6705802; doi:10.1371/journal.pone.0219765)
Supplement: S1 Fig — (DOCX) [file pone.0219765.s001.docx]

Supplemental figure S1. Oligonucleotides used in this study

Primers for quantitative PCR (qPCR)

| GENE | PRIMER FORWARD | PRIMER REVERSE COMPLEMENTARY |
| --- | --- | --- |
| PvTET1A | TCTCCAACCCTGCTTCACTCACT | CGGGATTGAGAGGATCACGG |
| PvTET1B | TTGGCAACCCTTAGGGTGGAG | CGATGTTCTGAATCAAGAGTAACCTTGC |
| PvTET2A | GGCGTGTCCATTTCCGTG | GAAGGAACTCACTTTGAGGGAGAC |
| PvTET3 | GCAAGCCCCCCACAGAATG | ACTTGCCAGCACACCAGCC |
| PvTET4 | TCCAACGGCACAGAATGCC | GATGAATCCGAGGAACACCAGC |
| PvTET5 | GTGAGAAGGGTGAGAGAGGTCAAG | CCAGCCCCAATGATGGGT |
| PvTET6 | AAGGAGGGAAGAAGGGTAGATTCG | CTGCTTCTCCTGCTGTCTTCAGC |
| PvTET8 | CCTCCAACGGCGAAATCG | TTTGTGTCTGTGGGCGCG |
| PvTET7 | GGCAGGGTGGGTTGAGAAGA | CACCCAAAACGATGAAGACTCTCTC |
| PvTET10 | AGCCCCAACAATGACTGCAAG | GATGGCTTCATGCTGCTTCTC |
| PvTET11 | CGACAGCAACCCACATAACTGC | GCGAATGCAAGGTACGAGGA |
| PvTET12 | TCGTGTCGGTTGGTATCCCTTTGTG | CAGCGTGTGGAGGATTCCGAC |
| PvEF1α | GGTCATTGGTCATGTCGACTCTGG | GCACCCAGGCATACTTGAATGACC |
| PvENOD40 | AGTTTTGTTGGCAAGCATCC | TAAGCACAAGCAAACTGTTG |
| PvRbOHB | GGAAGGAGATGCTCGATCTGC | GTCTTCACCCTTGTCCCTGAAAC |
| PvRabA2 | TCGCAGAAGACAATCCCCAC | CTGATCGATTATGACGCTACACAAA |
| PvPT4 | GCGGTGACTAACATGTTAGGG | CCTGTGCCCTAGTATTGTTGG |

Primers for amplifying CDS.

| GENE | PRIMER FORWARD | PRIMER REVERSE COMPLEMENTARY |
| --- | --- | --- |
| PvTET3 | **CACC**CTTCATCTCTCATGGTTCCAACAC | AAGATGAAAGGCACTAGGTTGTGC |
| PvTET6 | CACCTGAAGACAGCAGGAGAAGCAGC | ATAAAGTTGTTCTTTTCTGTCATGTAACCA |
| PvTET10 | CACCCAAGTTTTCCCTCTGCGGAA | AGCTTTAGAATGCCTCCTGGC |

Primers for amplifying the promoter region.

| GENE | PRIMER FORWARD | PRIMER REVERSE COMPLEMENTARY |
| --- | --- | --- |
| PvTET8 | CACCGGATATGTTAGGGTTGTAGTTCG | GAACCATGGTGAAGAAAGTGTCTTGGG |
| PvTET3 | CACCCTTTCCACTCCTCTCGTCTTTG | CTCATCTTGCAATCTTCTTCGCC |
| PvTET1 | CACC CTG TAT CAT TAT GCG TCACTGTAGC | GCC ATC TTG GTA GCA GAA ATG |
