# Supplementary figures and images for: Differential tetraspanin genes expression and subcellular localization during mutualistic interactions in Phaseolus vulgaris
Source: PLoS One. 2019 Aug 22;14(8):e0219765. doi: 10.1371/journal.pone.0219765 (PMC6705802; doi:10.1371/journal.pone.0219765)

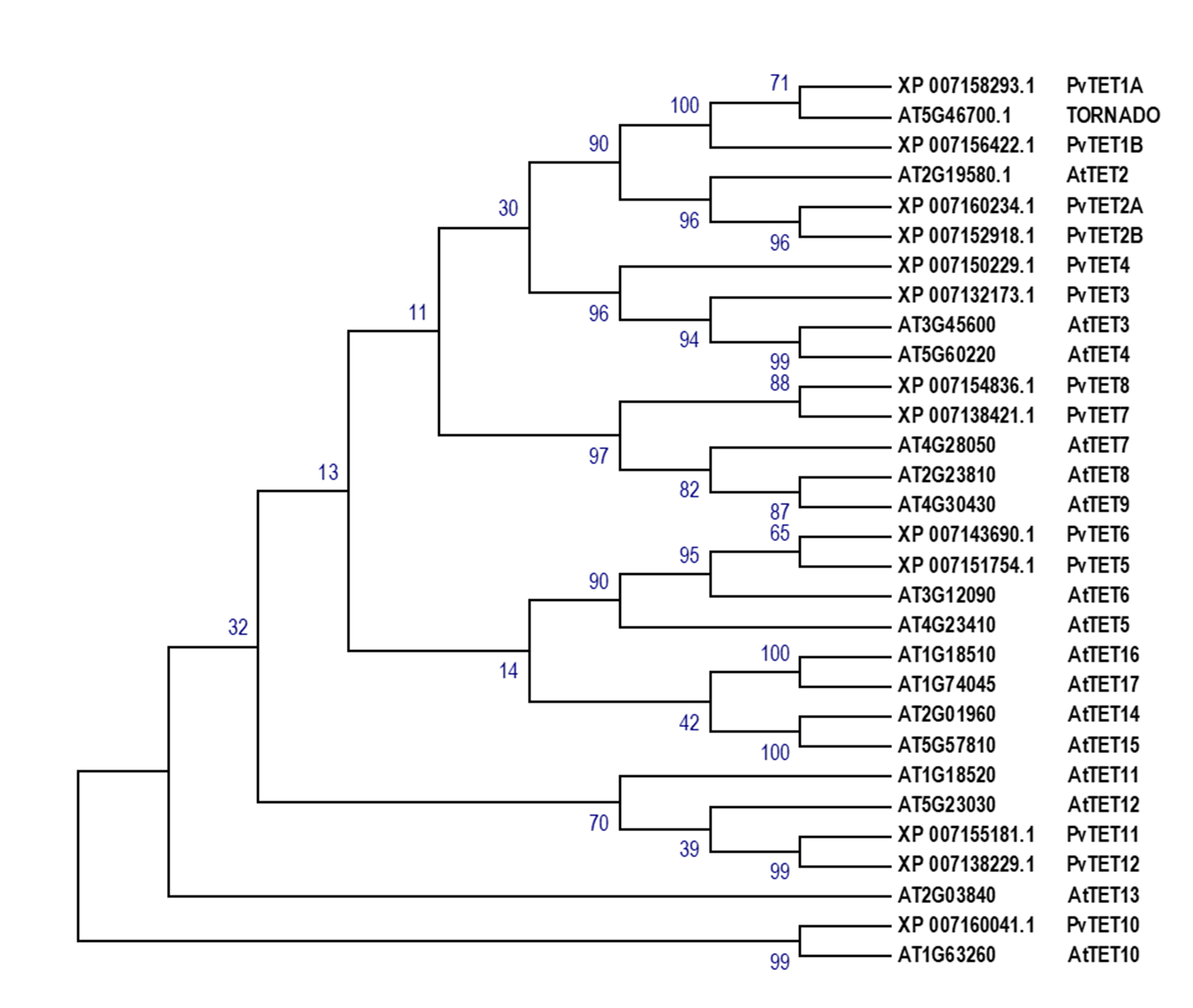

Supplement: S2 Fig — The phylogenetic tree was generated from the alignment of tetraspanin proteins with n = 1000 bootstrap replicates. The TET proteins were classified into clades based on phylogenetic analysis using the neighbor-joining (NJ) method. We used as query all tetraspanins reported by Boavida et al., 2013. (TIF) [file pone.0219765.s002.tif]

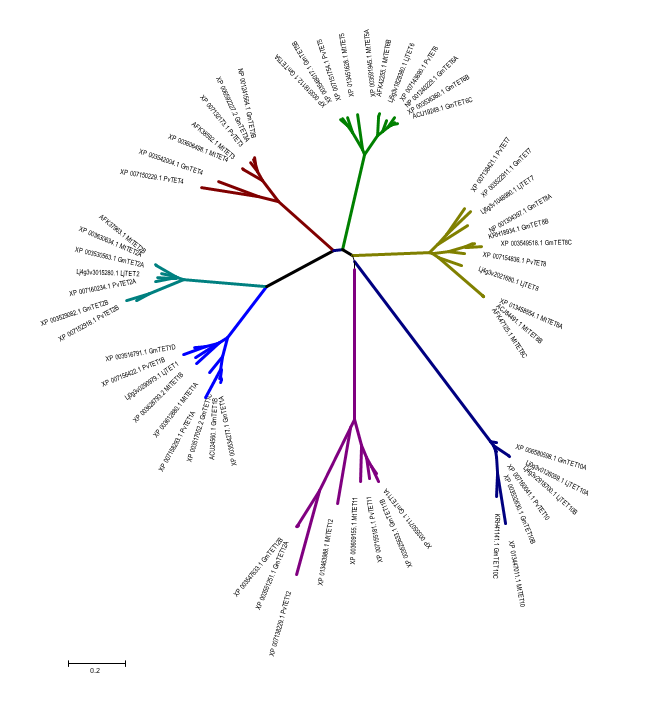

Supplement: S3 Fig — We selected amino acid sequences from Medicago truncatula, Phaseolus vulgaris, Glycine max, and Lotus japonicus. In this phylogenetic tree we schematize seven groups formed with legumes tetraspanin and are represented by different color branch. We selected a bootstrapping method to build the phylogenetic tree with 1000 replicates using MEGA Version 6.0.6 (Tamura et al., 2013) (TIF) [file pone.0219765.s003.TIF]

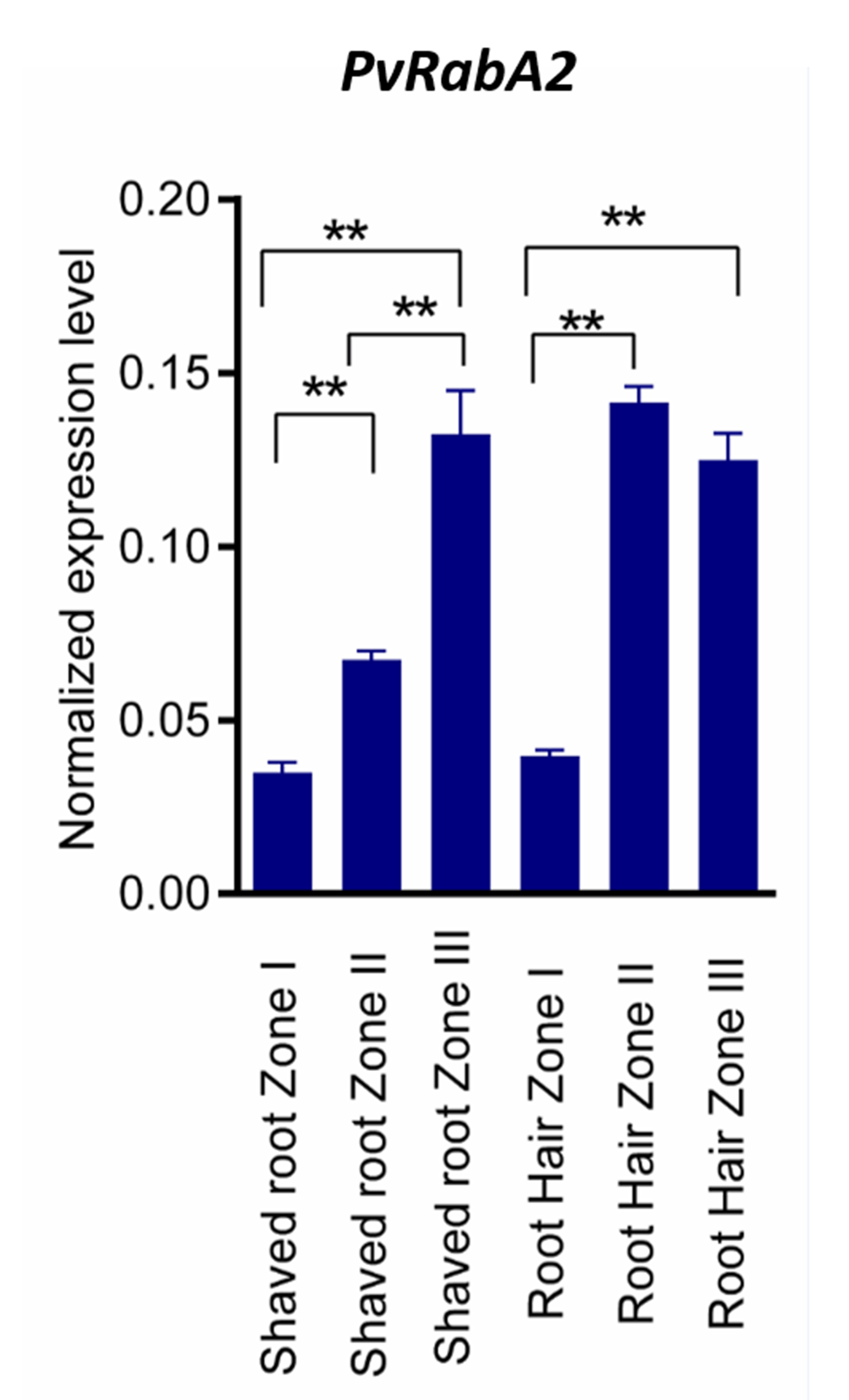

Supplement: S4 Fig — Transcript levels were quantified by reverse transcription and real-time PCR (RT-qPCR) and calculated using the expression levels of Elongation Factor 1α as reference. Measures were performed in each enriched tissues and different zones in root of common bean at 48 hpg. The number of biological replicates (n = 3) is indicated. Error bars indicate mean and SEM (±SEM). (TIF) [file pone.0219765.s004.tif]

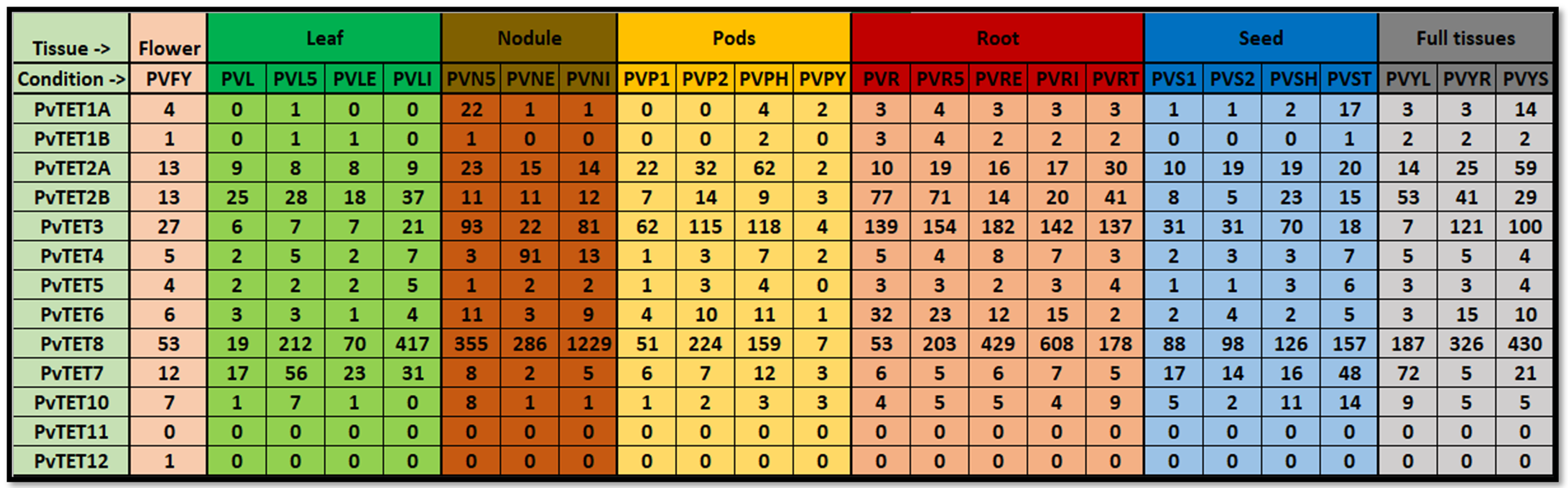

Supplement: S5 Fig — FY- Young flowers, collected prior to floral emergence; LF- Leaf tissue from fertilized plants collected at the same time of LE and LI; L5- Leaf tissue collected 5 days after plants were inoculated with effective rhizobium; LE- Leaf tissue collected 21 days after plants were inoculated with effective rhizobium; LI- Leaf tissue collected 21 days after plants were inoculated with ineffective rhizobium; N5- Pre-fixing (effective) nodules collected 5 days after inoculation; NE- Effectively fixing nodules collected 21 days after inoculation; NI- Ineffectively fixing nodules collected 21 days after inoculation; P1- Pods between 10 and 11 cm long, associated with stage 1 seeds (pod only); P2- Pods between 12 and 13 cm long associated with stage 2 seeds (pod only); PH- Pods approximately 9cm long, associated with seeds at heart stage (pod only); PY- Young pods, collected 1 to 4 days after floral senescence. Samples contain developing embryos at globular stage; R- Whole roots from fertilized plants collected at the same time as RE and RI; R5- Whole roots separated from 5 day old pre-fixing nodules; RE- Whole roots separated from fix+ nodules collected 21 days after inoculation; RI- Whole roots separated from fix- nodules collected 21 days after inoculation; RT- Root tips, 0.5 cm of tissue, collected from fertilized plants at 2nd trifoliate stage of development.; S1- Stage 1 seeds, between 6 and 7 mm across and approximately 50 mg; S2- Stage 2 seeds, between 8 and 10 mm across and between 140 and 150 mg; SH- Heart stage seeds, between 3 and 4 mm across and approximately 7 mg; ST- Shoot tip, including the apical meristem, collected at the 2nd trifoliate stage; YL- Fully expanded 2nd trifoliate leaf tissue from plants provided with fertilizer; YR- Whole roots, including root tips, collected at the 2nd trifoliate stage of development; YS- All stem internodes above the cotyledon collected at the 2nd trifoliate stage. Common bean atlas source (https://plantgrn.noble.org/PvGEA/blas [file pone.0219765.s005.tif]

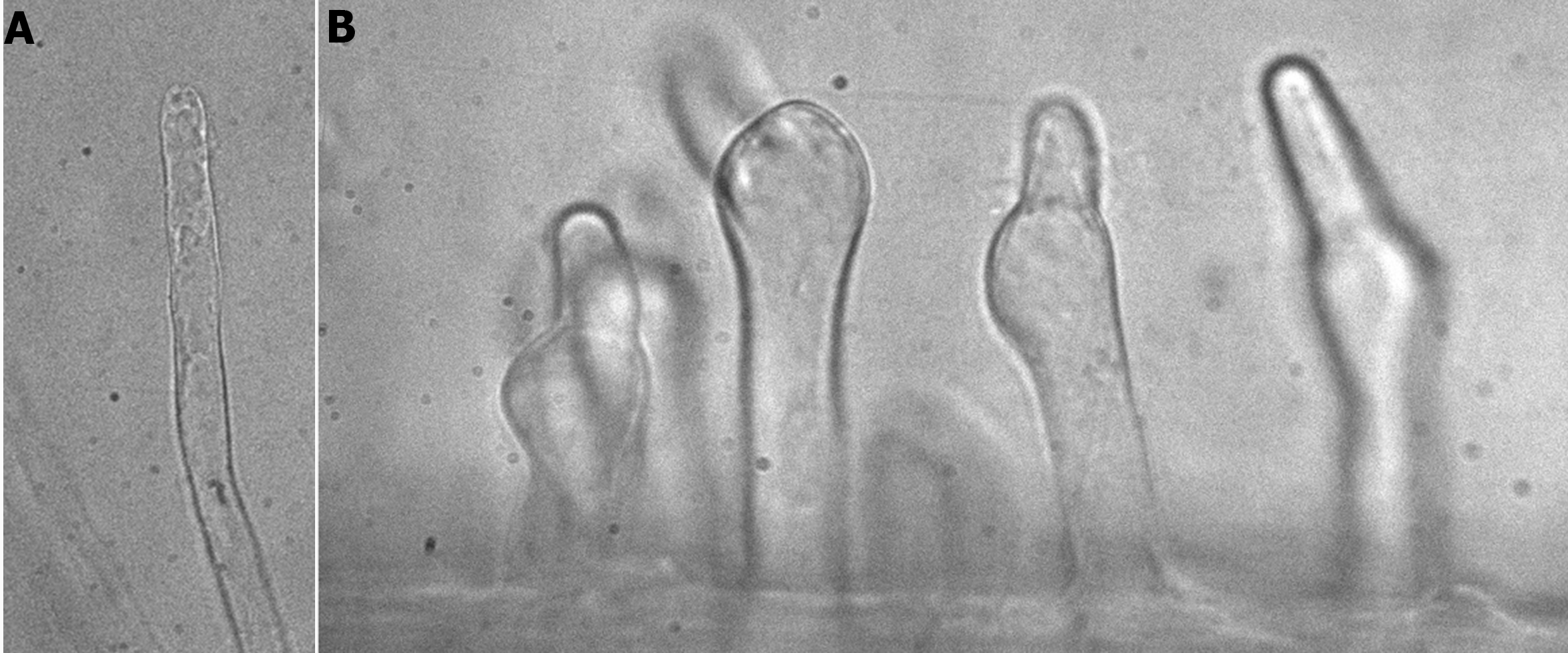

Supplement: S6 Fig — (A) Representative image of root hairs of roots of common bean under control condition treated with chitosan 10−9 M and (B) root hair subjected to a treatment with 10−9 M of NFs for 4 h (Scale = 100 μm). (TIF) [file pone.0219765.s006.tif]

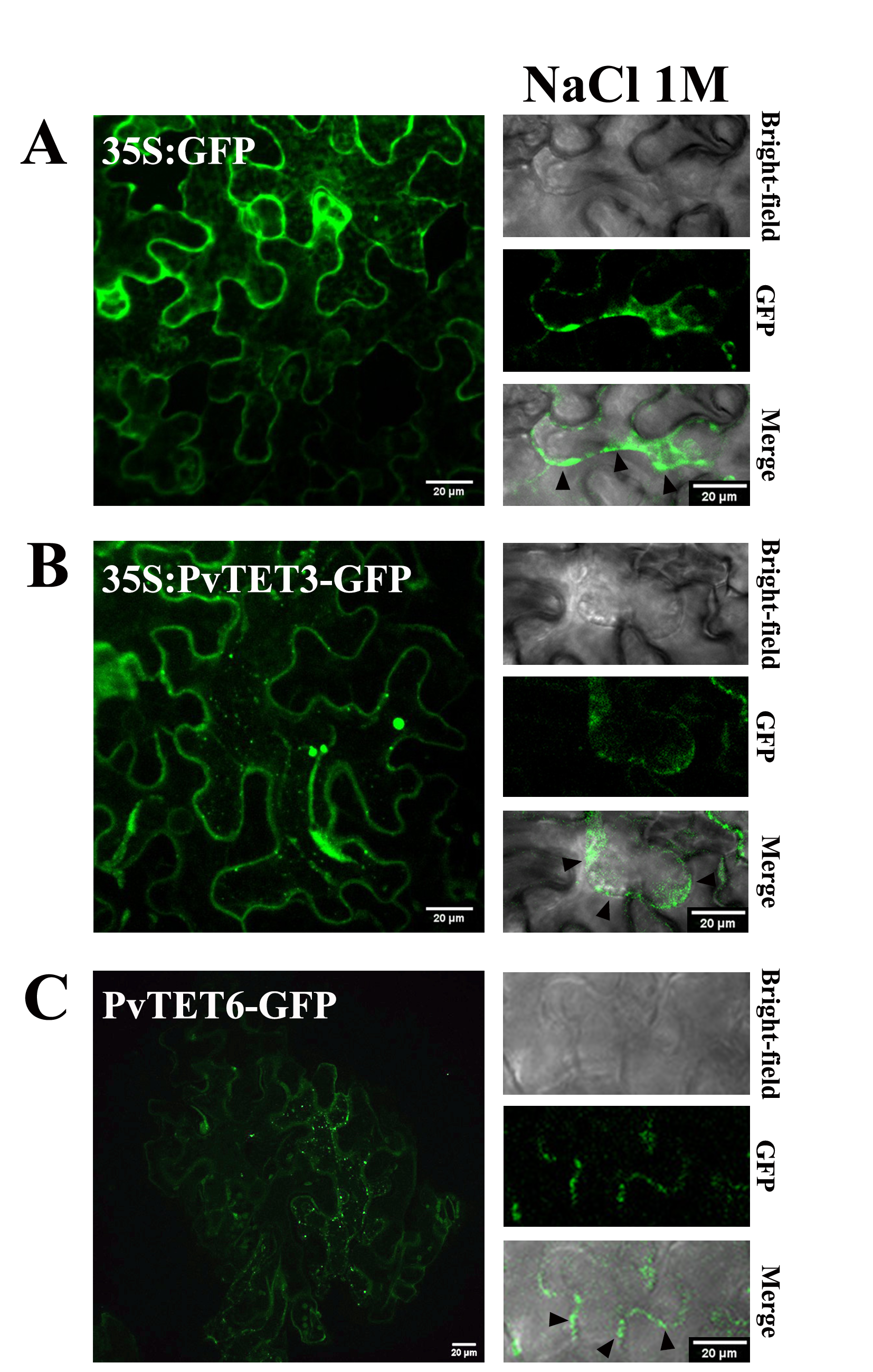

Supplement: S8 Fig — Agroinfiltrated cells from N. benthamiana leaves under plasmolysis induced by NaCl. A, B and C, 35S:GFP, 35S:PvTET3-GFP, 35S:PvTET6-GFP respectively, showing the regular cytoplasmic protein localization under control condition (left panel) and under plasmolysis (right small panels). Arrows in B and C indicates the 35S:PvTET3-GFP and 35S:PvTET6-GFP fluorescence associated with the retracted plasma membrane, while the 35S:GFP remains in the cytoplasm in A. (TIF) [file pone.0219765.s008.tif]
